# Association between Adult Height and Risk of Colorectal, Lung, and Prostate Cancer: Results from Meta-analyses of Prospective Studies and Mendelian Randomization Analyses

**DOI:** 10.1371/journal.pmed.1002118

**Published:** 2016-09-06

**Authors:** Nikhil K. Khankari, Xiao-Ou Shu, Wanqing Wen, Peter Kraft, Sara Lindström, Ulrike Peters, Joellen Schildkraut, Fredrick Schumacher, Paolo Bofetta, Angela Risch, Heike Bickeböller, Christopher I. Amos, Douglas Easton, Rosalind A. Eeles, Stephen B. Gruber, Christopher A. Haiman, David J. Hunter, Stephen J. Chanock, Brandon L. Pierce, Wei Zheng, on behalf of the Colorectal Transdisciplinary Study (CORECT), Discovery, Biology, and Risk of Inherited Variants in Breast Cancer (DRIVE), Elucidating Loci Involved in Prostate Cancer Susceptibility (ELLIPSE), Transdisciplinary Research in Cancer of the Lung (TRICL)

**Affiliations:** 1 Division of Epidemiology, Department of Medicine, Vanderbilt Epidemiology Center, Vanderbilt-Ingram Cancer Center, Vanderbilt University School of Medicine, Nashville, Tennessee, United States of America; 2 Program in Genetic Epidemiology and Statistical Genetics, Harvard T.H. Chan School of Public Health, Boston, Massachusetts, United States of America; 3 Public Health Sciences Division, Fred Hutchinson Cancer Research Center, Seattle, Washington, United States of America; 4 Cancer Prevention, Detection & Control Research Program, Duke Cancer Institute, Durham, North Carolina, United States of America; 5 Department of Preventive Medicine, Keck School of Medicine, University of Southern California/Norris Comprehensive Cancer Center, Los Angeles, California, United States of America; 6 Tisch Cancer Institute and Institute for Translational Epidemiology, Icahn School of Medicine at Mount Sinai, New York, New York, United States of America; 7 Division of Cancer Genetics/Epigenetics, Department of Molecular Biology, University of Salzburg, Salzburg, Austria; 8 Division of Epigenomics and Cancer Risk Factors, German Cancer Research Center (DKFZ), Heidelberg, Germany; 9 Translational Lung Research Center Heidelberg, German Center for Lung Research (DZL), Heidelberg, Germany; 10 Department of Genetic Epidemiology, University Medical Center, Georg-August-Universität Göttingen, Göttingen, Germany; 11 Center for Genomic Medicine, Geisel School of Medicine, Dartmouth College, Lebanon, New Hampshire, United States of America; 12 Centre for Cancer Genetic Epidemiology, Department of Oncology, University of Cambridge, Cambridge, United Kingdom; 13 Institute of Cancer Research, London, United Kingdom; 14 Royal Marsden NHS Foundation Trust, London, United Kingdom; 15 USC Norris Comprehensive Cancer Center, University of Southern California, Los Angeles, California, United States of America; 16 Department of Preventive Medicine, Keck School of Medicine, University of Southern California, Los Angeles, California, United States of America; 17 Department of Epidemiology, Harvard T.H. Chan School of Public Health, Boston, Massachusetts, United States of America; 18 Division of Cancer Epidemiology and Genetics, National Cancer Institute, National Institutes of Health, Bethesda, Maryland, United States of America; 19 Department of Public Health Studies, University of Chicago, Chicago, Illinois, United States of America; McGill University, CANADA

## Abstract

**Background:**

Observational studies examining associations between adult height and risk of colorectal, prostate, and lung cancers have generated mixed results. We conducted meta-analyses using data from prospective cohort studies and further carried out Mendelian randomization analyses, using height-associated genetic variants identified in a genome-wide association study (GWAS), to evaluate the association of adult height with these cancers.

**Methods and Findings:**

A systematic review of prospective studies was conducted using the PubMed, Embase, and Web of Science databases. Using meta-analyses, results obtained from 62 studies were summarized for the association of a 10-cm increase in height with cancer risk. Mendelian randomization analyses were conducted using summary statistics obtained for 423 genetic variants identified from a recent GWAS of adult height and from a cancer genetics consortium study of multiple cancers that included 47,800 cases and 81,353 controls. For a 10-cm increase in height, the summary relative risks derived from the meta-analyses of prospective studies were 1.12 (95% CI 1.10, 1.15), 1.07 (95% CI 1.05, 1.10), and 1.06 (95% CI 1.02, 1.11) for colorectal, prostate, and lung cancers, respectively. Mendelian randomization analyses showed increased risks of colorectal (odds ratio [OR] = 1.58, 95% CI 1.14, 2.18) and lung cancer (OR = 1.10, 95% CI 1.00, 1.22) associated with each 10-cm increase in genetically predicted height. No association was observed for prostate cancer (OR = 1.03, 95% CI 0.92, 1.15). Our meta-analysis was limited to published studies. The sample size for the Mendelian randomization analysis of colorectal cancer was relatively small, thus affecting the precision of the point estimate.

**Conclusions:**

Our study provides evidence for a potential causal association of adult height with the risk of colorectal and lung cancers and suggests that certain genetic factors and biological pathways affecting adult height may also affect the risk of these cancers.

## Abstract 2

In a Mendelian randomisation study Pierce and colleagues show a genetic association between adult height and increased risk of colorectal and lung cancer.

## Author Summary

**Why Was This Study Done?:**

- Several previous observational studies have examined the association between adult height and risk of cancers of the lung, colon/rectum, and prostate; however, it remains unclear whether adult height is indeed related to the risk of these cancers.

**What Did the Researchers Do and Find?:**

- We conducted a systematic review and meta-analysis of prospective cohort studies that examined the association between adult height and the risk of colorectal, lung, and prostate cancers.
- To overcome inherent limitations of observational study designs, we conducted Mendelian randomization analyses using genetic data generated from a large multi-center consortium study including 47,800 cases and 81,353 controls.
- In the meta-analysis of the prospective observational studies, we found a 12% increased risk of colorectal cancer, a 7% increased risk of prostate cancer, and a 6% increased risk of lung cancer for every ten-centimeter increase in height, and this increased risk was corroborated in the Mendelian randomization analyses for colorectal (58%) and lung cancer (10%).

**What Do These Findings Mean?:**

- Our study provides strong evidence for an association between adult height and risk of colorectal and lung cancer, and suggests that certain genetic and biological factors that affect height may also affect the risk of these cancers.
- However, our meta-analysis was limited to published studies, and the sample size for the Mendelian randomization analysis for colorectal cancer was relatively small, affecting the precision of the risk estimate.

## Introduction

Numerous studies have examined the relation between height and cancer; however, results have been inconsistent. The inconsistencies in observational studies could be due to factors that affect validity, including confounding, selection bias, reverse causation, and measurement error. Therefore, no consensus has been reached on whether height is a risk factor for colorectal, lung, or prostate cancer, three of the most common cancers affecting men and women. Furthermore, meta-analyses of prospective cohort studies, summarizing the association between height and colorectal, lung, or prostate cancers, have not been conducted.

Mendelian randomization is based on the principle that an individual’s genotype is randomized at conception [1] and utilizes genetic variants as instrumental variables for phenotypic exposures. This approach circumvents threats to validity found in conventional observational epidemiologic studies and potentially allows for causal inferences regarding the relation between exposure and disease [1–3]. Mendelian randomization analyses make the following assumptions regarding the genetic variants used in instrumental variables: (1) the genetic variants are associated with the exposure, (2) the genetic variants affect the outcome only via the exposure (also known as the “exclusion restriction”), and (3) the genetic variants are not associated with any confounders of the exposure-outcome association [4]. A recent genome-wide association study (GWAS) identified nearly 700 variants—reflecting 423 loci—that were associated with adult height in individuals of European descent [5]. These variants explain approximately 16% of height variance and have the potential to serve as strong instruments in Mendelian randomization analyses.

To comprehensively evaluate the association between height and risk of colorectal, lung, and prostate cancers, we conducted a systematic review and meta-analysis of previous prospective studies. Additionally, we carried out Mendelian randomization analyses utilizing GWAS summary statistics from the Genetic Associations and Mechanisms in Oncology (GAME-ON) and Genetic Investigation of Anthropometric Traits (GIANT) consortia studies of individuals of European descent.

## Methods

### Systematic Review and Meta-analysis

We searched PubMed, Embase, and Web of Science on January 3, 2016, for prospective studies (i.e., prospective cohort, nested case-control, and case-cohort studies) using the following search terms: lung neoplasms; lung cancer; colorectal neoplasms; colorectal cancer; prostate neoplasms; prostate cancer; lung, prostate, colorectal, colon, rectum, rectal or cancer; and body height, height, stature, body size, anthropometrics, or anthropometry. After restricting the results to English language and humans, a total of 15,691 publications were found.

**Fig 1:**
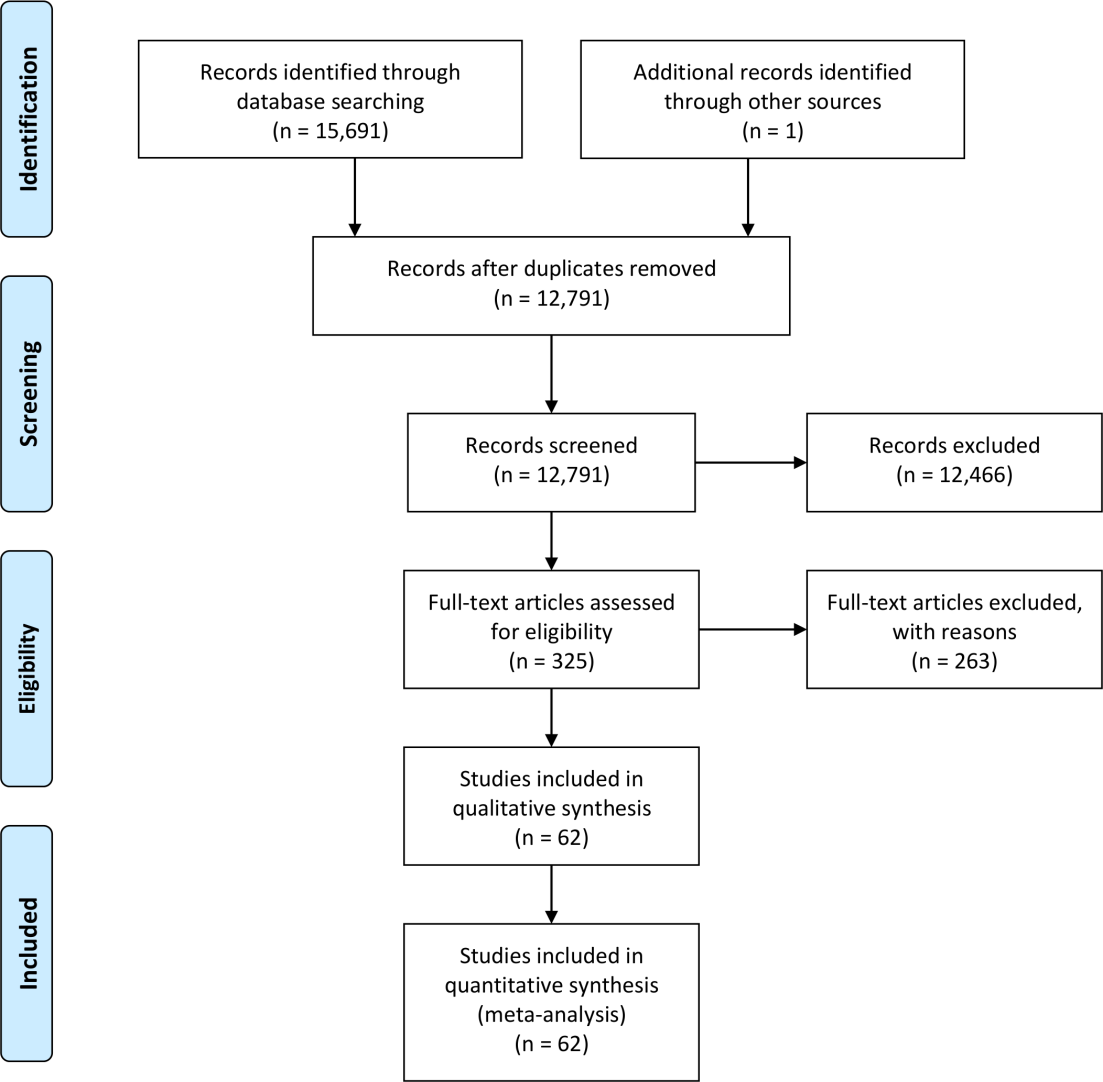
PRISMA flow chart for studies included in the meta-analysis.

Approximately 2,900 publications were duplicates. Following the title and abstract review, additional exclusions for outcomes other than cancer resulted in a total of 325 studies for full-text review. One Chinese study, for which all relevant tables and methods were written in English, was additionally included [6]. Full-text review revealed height examined in relation to lung cancer in 11 studies [6–16], to colorectal cancer in 24 studies [6,9–31], and to prostate cancer in 27 studies [6,9–11,16,30,32–52]; these studies were included in the meta-analysis. We also conducted a meta-analysis for a subset of prostate cancer studies that also reported estimates for aggressive prostate cancer (defined as Gleason score ≥ 7, metastatic spread, or regional/distant stage) [40,41,43–52]. Given that several studies examined multiple cancer sites, the final set of studies for each cancer type included in the meta-analysis is not exclusive. Fig 1 summarizes the search results and exclusions for this systematic review and the final set of studies used in the meta-analysis (detailed diagram according to cancer site provided in S5 Fig). Details regarding each study included in the meta-analysis are provided in S1–S4 Tables. We recently published a large-scale meta-analysis of prospective studies of the association of height with breast cancer (more than 113,000 incident breast cancers) [53]; we present the meta-analysis results for breast cancer from our previous study for comparison.

All study estimates were scaled for a 10-cm increase in height in relation to cancer, which, in general, approximated the average interquartile range in height across all studies. For studies that reported categorical estimates of height (i.e., quantiles), a continuous estimate and standard error was estimated using the method proposed by Greenland and Longnecker [54] using R package “dosresmeta” (R version 3.1.2). Scores were generated for each quantile for studies reporting categorical estimates and were equivalent to either the mean or median; scores for the uppermost open-ended quantiles were generated using the method presented by Il’yasova et al. [55].

Study-specific estimates were summarized using inverse-variance-weighted (IVW) fixed- and random-effects meta-analyses for all three cancers (colorectal, lung, and prostate) separately. Fixed-effects models assume no heterogeneity between the study estimates, whereas random-effects models take heterogeneity of the study estimates into consideration. Cochran’s test for homogeneity was applied in the analysis, with *p* < 0.05 indicating heterogeneity among the studies. Summary odds ratios (ORs) and 95% CIs for the random-effects meta-analyses were estimated using restricted maximum likelihood. Funnel plots were generated for each cancer to examine publication bias and were formally assessed using Begg’s and Egger’s statistical tests (S1–S4 Figs); no violations were observed. Meta-analysis was conducted using Stata version 12.1.

### Mendelian Randomization Analysis Using Summary Statistics

Results from our recent Mendelian randomization analysis of adult height and breast cancer have been published [53], and thus the current analysis focuses on cancers of the colorectum, lung, and prostate. The analysis was conducted to estimate the effect of height (*X*) on the risk of cancer (*Y*) using genetic variants (*g*), where the causal estimate is equal to *Y*_*g*_ */X*_*g*_ [56]. For the association between genetic variants and height (*X*_*g*_), we utilized available summary statistics from the GIANT GWAS. Summary statistics for the association between genetic variants and each of the three cancers (*Y*_*g*_) are from the GAME-ON consortium.

Nearly 700 genetic variants from 423 loci were identified as reaching genome-wide significance (*p* < 5 × 10^−8^) in the GIANT consortium study, explaining 16% of phenotypic variation in height [5]. In our study, we selected 423 uncorrelated variants, representing these loci, to construct the instrumental variables in our Mendelian randomization analysis (S5 Table).

GAME-ON data were utilized to provide regression coefficients and standard errors for each single nucleotide polymorphism (SNP) in relation to cancer (*Y*_*g*_). GAME-ON is network of genetic epidemiology consortia that contains GWAS data from the Colorectal Transdisciplinary Study (CORECT); Discovery, Biology, and Risk of Inherited Variants in Breast Cancer (DRIVE); Elucidating Loci Involved in Prostate Cancer Susceptibility (ELLIPSE); Follow-up of Ovarian Cancer Genetic Association and Interaction Studies (FOCI); and Transdisciplinary Research in Cancer of the Lung (TRICL). For the current study, we used data from CORECT, ELLIPSE, and TRICL. The GAME-ON consortium primarily consists of studies of individuals of European descent. SNPs with poor imputation quality (*r* ^2^ < 0.3) were excluded (CORECT used IMPUTE 2.0 info score < 0.7 as the cutoff) [57]. The association between adult height and breast cancer risk was evaluated in our previous study using 168 height-associated variants [53]. For the analysis presented here, we updated the Mendelian randomization summary estimate for breast cancer using data from DRIVE for the 423 newly identified height-associated variants.

Not all SNPs with information regarding *X*_*g*_ (from the GIANT consortium) had corresponding information regarding *Y*_*g*_ (from GAME-ON); therefore, these SNPs were excluded from the instrumental variables for colorectal cancer (*n* = 77) and prostate cancer (*n* = 4). No SNPs were excluded from the instrument for lung cancer. Thus, there were fewer instrumental variables for the colorectal and prostate cancer studies than for the lung cancer study. Using both summary statistics for *Y*_*g*_ and *X*_*g*_, an IVW meta-analysis was conducted to estimate the effect of genetically determined height on the risk of each cancer using the method of Burgess et al. [56]:

$${\hat{\beta }}_{\text{IVW}}=\frac{\sum_{i=1}^{g}X_{g}Y_{g}\sigma_{Yg}{}^{\text{-}2}}{\sum_{i=1}^{g}X_{g}{}^{2}\sigma_{Yg}{}^{\text{-}2}},\;\text{se}\left({\hat{\beta }}_{\text{IVW}}\right)=\sqrt{\frac{1}{\sum_{i=1}^{g}X_{g}{}^{2}\sigma_{Yg}{}^{\text{-}2}}}$$

where *X*_*g*_ is the beta estimate for the association between the SNP and height (from GIANT), *Y*_*g*_ is the beta estimate for the association between the SNP and cancer (from GAME-ON), and σ_*Yg*_ is the standard error for *Y*_*g*_. Corresponding ORs and 95% CIs were calculated using ${\hat{\beta }}_{\text{IVW}}$ and $\text{se}\left({\hat{\beta }}_{\text{IVW}}\right)$. All ORs and 95% CIs were subsequently standardized per 10 cm. SAS version 9.4 was utilized for the Mendelian randomization analysis using summarized data.

### Sensitivity Analyses

We conducted several sensitivity analyses to assess whether the association of height with cancer varied for different subgroups. Using data from a previous GIANT height GWAS that presented sex-stratified summary statistics for 168 independent SNPs explaining approximately 10% of variation in height [58], we conducted Mendelian randomization sensitivity analyses for colorectal and lung cancers for males and females separately. Additionally, in order to account for potential pleiotropy of the SNPs utilized in the genetic instrument (potentially violating the exclusion restriction assumption for a valid instrumental variable), we excluded SNPs with suggested pleiotropic effects associated with various diseases and traits (e.g., pulmonary function, phospholipid levels, cardiovascular disease, and cancer) [5]. Additionally we used a data-driven approach to explore violations of pleiotropy using Egger regression [59]. For the meta-analysis, we conducted additional analyses stratified by cancer of the colon versus rectum and self-reported height versus measured height. Given that height is associated with lung size and lung function [60]—which could lead to efficient nicotine uptake [61] and possibly increased likelihood of smoking initiation [62] among taller individuals—we also stratified our meta-analysis results by studies that adjusted for potential confounding by smoking. Finally, we conducted sensitivity meta-analyses for colorectal, prostate, and lung cancers, including only those studies of populations of European descent.

## Results

### Meta-analysis

**Fig 2:**
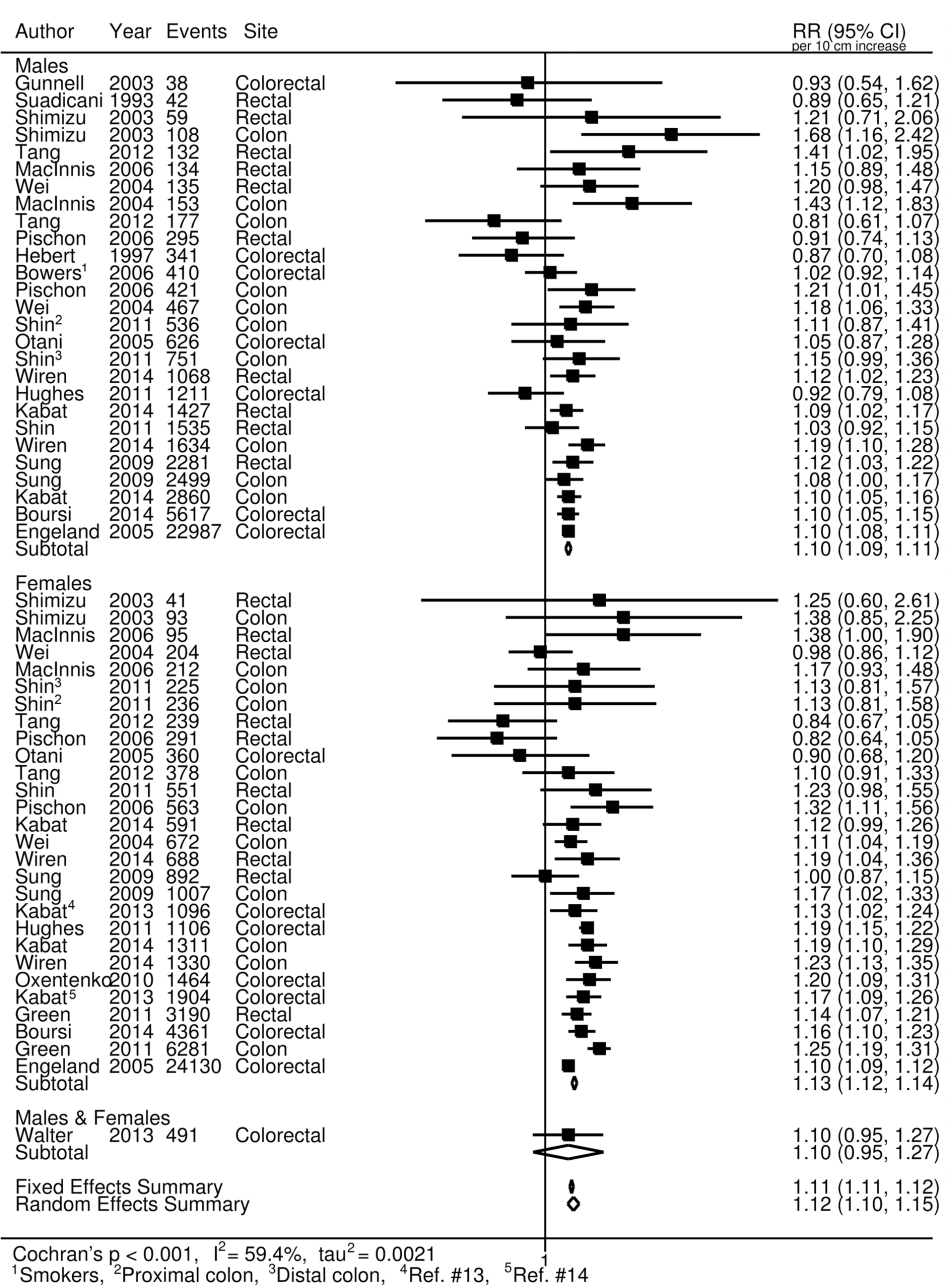
Forest plot for prospective studies of adult height and colorectal cancer, stratified by sex. RR, relative risk.

**Fig 3:**
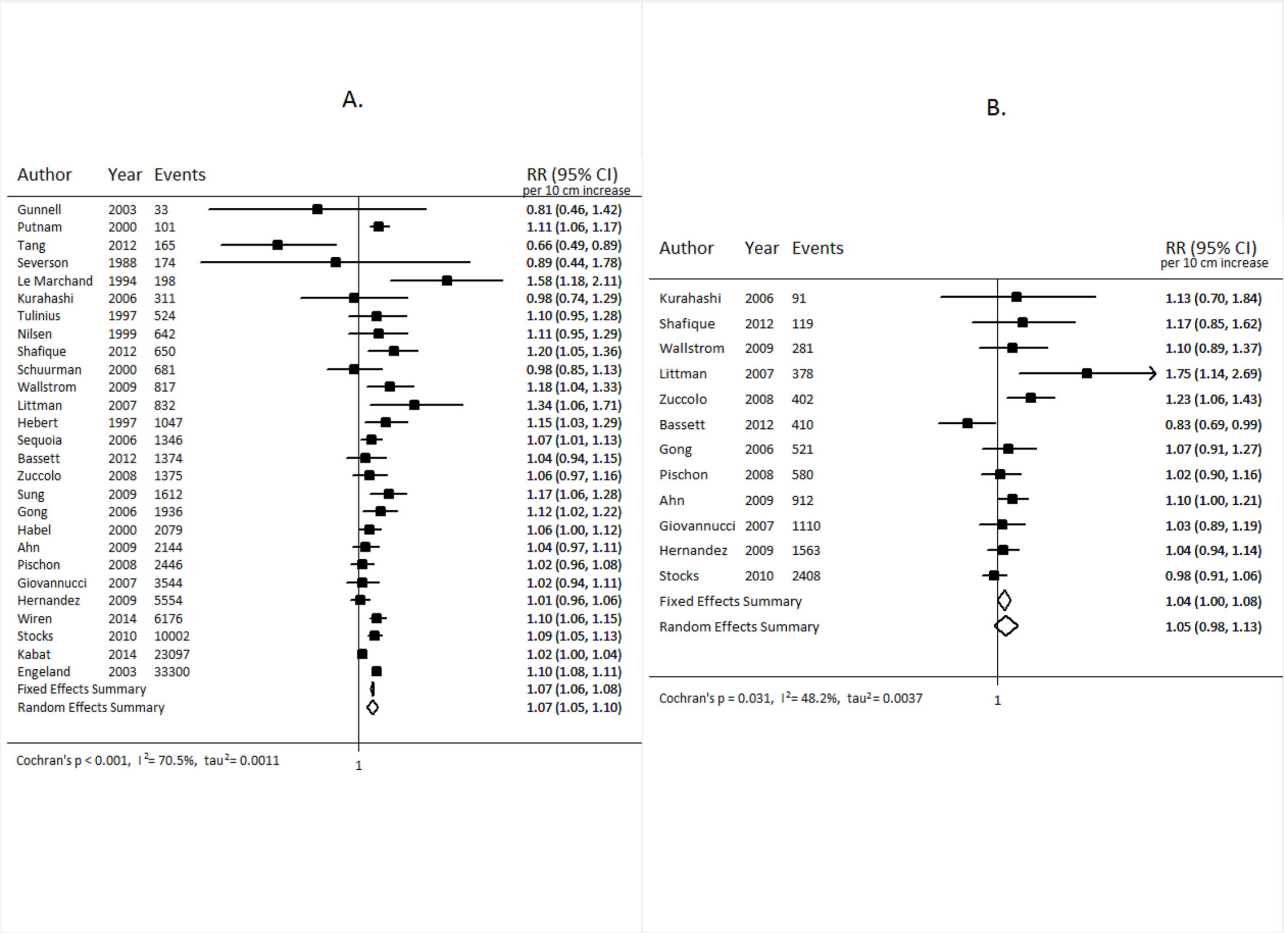
Forest plot for prospective studies of adult height and prostate cancer. Overall prostate cancer (A); aggressive prostate cancer (B). RR, relative risk.

**Fig 4:**
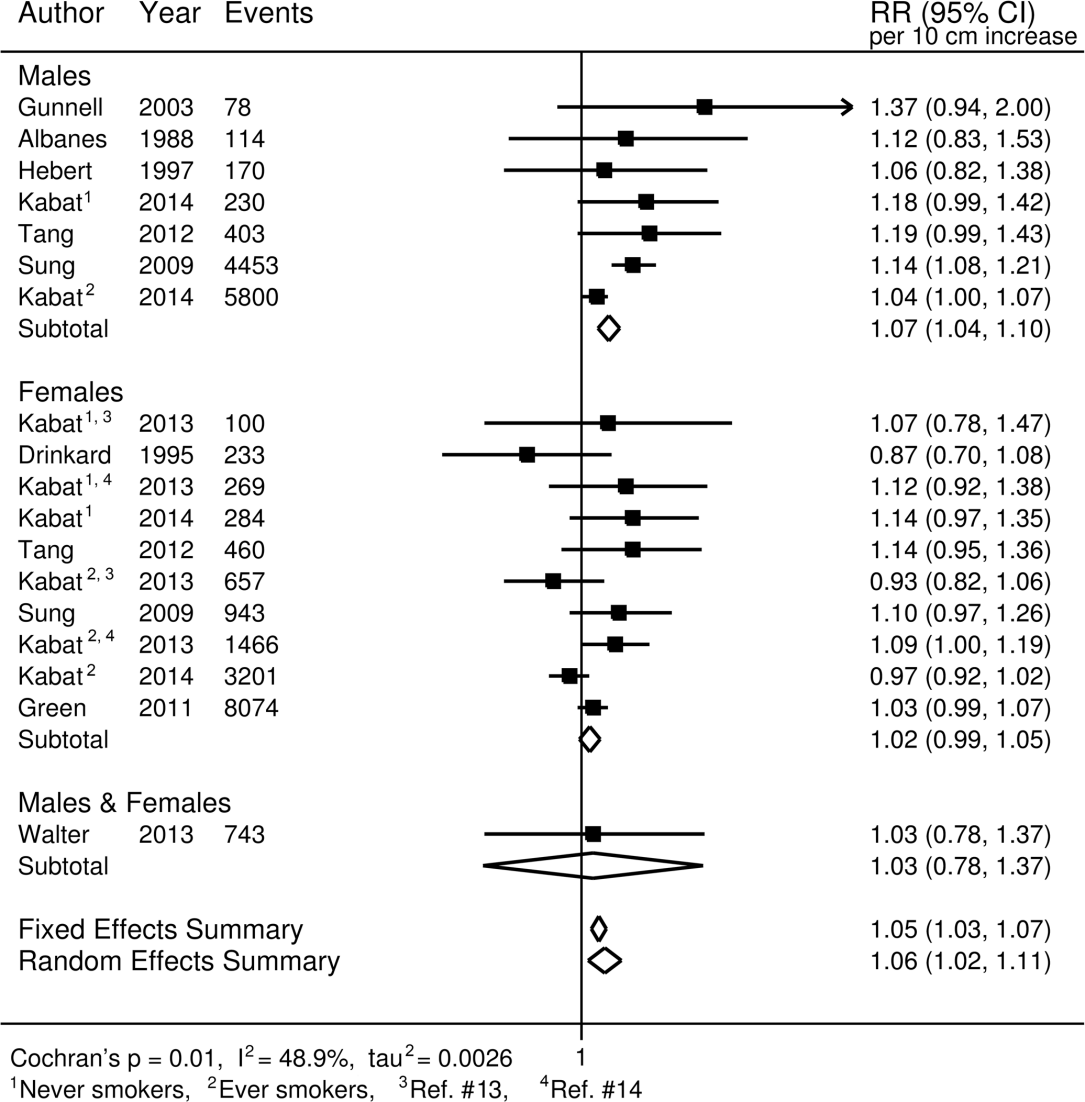
Forest plot of prospective studies of adult height and lung cancer, stratified by sex. RR, relative risk.

For colorectal cancer (Fig 2), we observed an approximately 12% increased risk (95% CI 1.10, 1.15) for a 10-cm increase in adult height (*p* < 0.001); no important differences were observed when stratified by sex. A 7% increase (95% CI 5%, 10%) in the risk of prostate cancer (*p* < 0.001; Fig 3A), and a 5% increase (95% CI −2%, 13%) in the risk of aggressive prostate cancer, was observed for a 10-cm increase in height (*p* = 0.031; Fig 3B). A 10-cm increase in height was associated with a 7% increase in lung cancer risk in males (relative risk [RR] = 1.07, 95% CI 1.04, 1.10; Fig 4) and a 2% increase in females (RR = 1.02, 95% CI 0.99, 1.05; Fig 4).

In general, the results from our sensitivity analyses, stratifying the studies based on several different factors, were not substantially different from those of the main analysis. After stratification by cancer site, our meta-analysis random-effects summary estimate for rectal cancer was lower (RR = 1.10, 95% CI 1.06, 1.13) than that for colon cancer (RR = 1.17, 95% CI 1.14, 1.19). No apparent differences were observed for lung cancer between studies that adjusted for smoking (RR_smoking-adjusted_ = 1.06, 95% CI 1.00, 1.11) and those that did not (RR_not-adjusted_ = 1.08, 95% CI 0.98, 1.21). Similarly, no differences were observed for smoking-adjusted studies for colorectal cancer (RR_smoking-adjusted_ = 1.11, 95% CI 1.08, 1.15; RR_not-adjusted_ = 1.13, 95% CI 1.09, 1.17). For prostate cancer, a modest difference was observed (RR_smoking-adjusted_ = 1.05, 95% CI 0.98, 1.12; RR_not-adjusted_ = 1.10, 95% CI 1.08, 1.11; homogeneity *p* = 0.181), but the difference was not statistically significant. Studies using self-reported height and studies using measured height yielded very similar summary estimates for prostate cancer (RR_self-reported_ = 1.07, 95% CI 1.00, 1.14; RR_measured_ = 1.09, 95% CI 1.07, 1.11) and colorectal cancer (RR_self-reported_ = 1.13, 95% CI 1.08, 1.18; RR_measured_ = 1.12, 95% CI 1.09, 1.14). However, some modest differences were observed for lung cancer (RR_self-reported_ = 1.02, 95% CI 0.98, 1.07; RR_measured_ = 1.10, 95% CI 1.04, 1.17; homogeneity *p* = 0.062). Additionally, the random-effects summary estimates for colorectal, prostate, and lung cancers did not differ substantially when we restricted the studies to only those conducted among populations of European descent (S6 Table).

### Mendelian Randomization Analysis

Results from the Mendelian randomization analyses are presented in Table 1. A nearly 60% increased risk of colorectal cancer was observed per 10-cm increase in genetically predicted height (OR = 1.58, 95% CI 1.14, 2.18, *p* = 0.006); males and females had slightly different estimates per 10-cm increase in height, with females having a slightly higher risk than males (S7 Table). A modest association with genetically predicted height was observed for lung cancer (OR = 1.10, 95% CI 1.00, 1.22, *p* = 0.053). The association appears to be stronger for adenocarcinoma of the lung (OR = 1.16, 95% CI 1.00, 1.35, *p* = 0.056) than for squamous cell carcinoma of the lung (OR = 1.06, 95% CI 0.90, 1.25, *p* = 0.463), and the homogeneity *p*-value was not statistically significant (*p* = 0.427). However, the estimates for males and females according to subtype were nearly identical (S7 Table). No statistically significant association with genetically predicted height was seen for prostate cancer (overall prostate cancer, OR = 1.03, 95% CI 0.92, 1.15, *p* = 0.642; aggressive prostate cancer, OR = 0.98, 95% CI 0.84, 1.15, *p* = 0.822). The results for breast cancer showed an approximately 20% increase in risk per 10-cm increase in genetically predicted height (OR = 1.19, 95% CI 1.07, 1.33, *p* = 0.001), which is very similar to our previous estimate using a smaller set of height-associated genetic variants (OR = 1.21, 95% CI 1.05, 1.39, *p* = 0.008) [53].

**Table 1:** Odds ratios and 95% confidence intervals estimated from Mendelian randomization analyses compared to the summary estimates (Figs 2–4) from published prospective studies for the association between adult height and cancers of the breast, colorectum, prostate, and lung.

Cancer Site	Mendelian Randomization						Meta-analysis of Published Prospective Studies	
	Cases^a^	Controls^a^	IV^b^	OR^c^	95% CI	*p*-Value	RR^c^	95% CI
**Breast**								
Overall	16,003	46,525	423	1.19	1.07, 1.33	0.001	1.17^d^	1.15, 1.19
**Colorectal**								
Overall	5,100	4,831	346	1.58	1.14, 2.18	0.006	1.12	1.10, 1.15
**Prostate**								
Overall	14,160	12,724	419	1.03	0.92, 1.15	0.642	1.07	1.05, 1.10
Aggressive	4,446	12,724	419	0.98	0.84, 1.15	0.822	1.05	0.98, 1.13
**Lung**								
Overall	12,537	17,285	423	1.10	1.00, 1.22	0.053	1.06	1.02, 1.11
Adenocarcinoma	3,804	16,289	423	1.16	1.00, 1.35	0.056	na	na
Squamous cell carcinoma	3,546	16,434	423	1.06	0.90, 1.25	0.463	na	na

^a^Summary sample sizes of studies included in the GAME-ON consortium.

^b^The total number of SNPs used to construct the instrumental variable.

^c^OR and RR represent the risk associated with a 10-cm increase in adult height.

^d^Meta-analysis summary OR and 95% CI previously reported [53].

IV, instrumental variable; na, not available; OR, odds ratio; RR, relative risk.

We did not observe any substantial differences in the Mendelian randomization estimates after excluding genetic variants with suggested pleiotropic effects from the instrument (S8 Table). Furthermore, the impact of pleiotropy may be negligible, given that the average pleiotropic effect was small and the intercept from the data-driven Egger regression was not statistically significant (S9 Table).

## Discussion

In this meta-analysis involving 62 published prospective studies, we observed a statistically significant increased risk for colorectal, prostate, and lung cancers associated with higher adult height. Our Mendelian randomization analyses confirmed these increased risks for colorectal and lung cancers but not for prostate cancer, indicating perhaps that the increased risk reported from previous prospective cohort studies for prostate cancer may be due to biases. To our knowledge, this is the first meta-analysis performed to summarize results from prospective cohort studies regarding the association of adult height and these three major cancers that affect the lives of men and women throughout the world. To our knowledge, this is also the first Mendelian randomization analysis performed to evaluate the association between adult height and risk of lung cancer. Our study, using an instrument comprising 423 height-related SNPs and explaining approximately 16% of phenotypic variation, provides strong evidence for a possible causal association between adult height and risk of breast, colorectal, and lung cancers. It suggests that certain genetic factors and biological pathways affecting adult height may affect the risk of these major cancers.

One potential reason taller individuals may be at higher risk is the increased opportunity for a cell to mutate given that taller people have more cells. However, multiple interrelated biological pathways have also been implicated in the association between height and cancer. One pathway is the insulin-like growth factor (IGF) pathway, which is known to promote cell proliferation and inhibit apoptosis [63]; several genetic variants of the IGF pathway have been identified as being related to height. Growth hormone is also known to stimulate IGF-1 expression, which plays an important role in determining adult height via regulation of bone growth [64]. Thus, attained height may represent cumulative exposure to IGF-1 throughout important periods of growth and development (i.e., in utero, childhood, adolescence) [65]. High levels of IGF-1 have also been shown to be positively associated with colorectal, lung, and prostate cancers [66–68]. Other lifestyle risk factors for colorectal cancer, such as increased caloric intake [69], being overweight [70], and having a sedentary lifestyle [71], have demonstrated higher levels of IGF-1 via increased insulin production and subsequent inhibition of IGF-binding protein synthesis [70]. Therefore, it is possible that high IGF-1 may be one of the underlying biological mechanisms mediating the association of height and colorectal cancer.

It is unlikely that the IGF-1 pathway alone would explain entirely the observed increased colorectal and lung cancer risk associated with adult height. Other pathways have also been revealed recently to influence adult height, including transforming growth factor beta (TGF-β) [72] and Hedgehog [73,74]. A number of loci (*SMAD3*, *MTOR*, *GLI2*, *LAMA5*) involved in these pathways were related to height in a recent GWAS [5]. Some of these pathways have also been linked to the pathogenesis of colorectal and lung cancers.

The results are similar when comparing the summary estimates from the meta-analysis and the associations from the Mendelian randomization analysis for both breast and lung cancers. For colorectal cancer, however, the association from our Mendelian randomization analysis was substantially stronger than that estimated from the meta-analysis of prospective cohort studies. This difference is likely due to sampling errors, given the small sample size of our Mendelian randomization analysis performed for colorectal cancer. Indeed, in a recent Mendelian randomization study with a larger sample size (more than 10,000 cases and 10,000 controls) than our study, a 10-cm increase in height was associated with a 7% (95% CI 1%, 14%) increased risk of colorectal cancer, and the risk was higher among females (OR = 1.09, 95% CI 1.01, 1.19) than males (OR = 1.05, 95% CI 0.96, 1.15) [75]. Although the magnitudes of the associations were different, a similar pattern—where females had a slightly higher risk than males—was also observed in our analysis (S7 Table). The consistency of the association in these two studies regarding adult height and increased colorectal cancer risk provides further support for a possible causal association.

We report modest increases in the risk of lung cancer with height in both the meta-analysis of prospective studies and the Mendelian randomization analysis. The majority of lung cancers are caused by smoking, and smoking may reduce IGF-1 levels [66]. Future investigations using individual-level data may consider stratification by smoking status to assess whether or not the association of height and lung cancer may be modified by tobacco smoking. For prostate cancer, we observed a null association with height in the Mendelian randomization analysis, providing no support for the modestly elevated risk observed in previous prospective cohort studies between adult height and the risk of prostate cancer. Our results are supported by a recently published Mendelian randomization study utilizing individual-level data that reported a null association between adult height and prostate cancer risk [76]. Our study should have 80% statistical power to detect a 9% increased risk in all prostate cancer associated with adult height, and an 11% increased risk in aggressive prostate cancer [77]. Therefore, we could not exclude the possibility of a weak association of adult height with prostate cancer.

There are several strengths to this study. The comprehensive literature search identified 62 studies related to adult height and the cancers evaluated in our study. Additionally, comparing the results from the Mendelian randomization analysis with the meta-analysis summary estimates could indicate whether or not the results from observational studies are biased, assuming the Mendelian randomization analyses represent an effect that may be closer to the true effect. Furthermore, we believe that the genetic variants used in our Mendelian randomization analysis serve as an adequate instrument for height for several reasons. One of the assumptions for a valid instrument is that the genetic marker is associated with the exposure phenotype of interest [3]. In our analysis, we utilized multiple genetic variants explaining approximately 16% of variation in height. It has also been demonstrated that using multiple SNPs helps to strengthen the genetic instrument and improve the precision of the estimate [56], and reduces bias stemming from potential violations of the other Mendelian randomization assumptions [4]. Additionally, all of the variants utilized were uncorrelated, and those that were not genotyped directly were imputed with high accuracy (*r* ^2^ ≥ 0.3 for ELLIPSE and TRICL, and info score ≥ 0.7 for CORECT) [57]. We also explored the influence of pleiotropy by excluding variants (*n* = 36) with suggested pleiotropic effects on several different biological outcomes, including pulmonary function, sex hormone binding globulin levels, and age at menarche. Pleiotropic effects would violate the exclusion restriction assumption for a valid instrument, which states that the instrument affects the outcome only through the exposure phenotype of interest [3,4]. However, as our results from the sensitivity analyses indicate, removing the pleiotropic SNPs from the instrument did not appreciably alter the results. Furthermore, the average pleiotropic effect, estimated using a data-driven approach via Egger regression [59], was not statistically significant.

Our study also has several limitations. First, the meta-analyses for the three different cancers are subject to the limitations of the original epidemiologic studies, which could include measurement errors, confounding, selection bias, and random error. Additionally, concerns regarding causal inference from Mendelian randomization studies remain, given the strict criteria required for a valid instrument. In particular, we could not entirely exclude the possible influence of pleiotropic effects on our results since some of the SNPs used in our study might be associated with certain unknown traits that may be related to cancer risk. However, such an influence, if it exists, should be very small, since an instrumental variable constructed using more than 400 height-associated SNPs should be much more strongly associated with adult height than with any other traits. The sample sizes for the analysis of colorectal cancer and for the subgroup analyses of prostate and lung cancers are relatively small, and the relationship between height and these cancers warrants further study using larger sample sizes. Given that most studies showed a linear association between adult height and cancer risk, we assumed a linear association in this analysis, which helped in harmonizing estimates and allowed us to conduct a comprehensive meta-analysis including all relevant studies. A formal test of the shape of the association may be needed in future studies.

In summary, this large-scale meta-analysis of prospective studies and Mendelian randomization analysis provide strong evidence for a possible causal association between adult height and the risk of colorectal and lung cancers. Our study suggests that certain biological pathways affecting adult height may also be involved in the etiology of both colorectal and lung cancers.

## Supporting Information

S1 Fig Funnel plot with pseudo 95% confidence limits for prospective studies of height and colorectal cancer (Begg’s *p-*value = 0.596, Egger’s *p-*value = 0.570). (EPS)

S2 Fig Funnel plot with pseudo 95% confidence limits for prospective studies of height and prostate cancer (Begg’s *p-*value = 0.835, Egger’s *p-*value = 0.930). (EPS)

S3 Fig Funnel plot with pseudo 95% confidence limits for prospective studies of height and aggressive prostate cancer (Begg’s *p-*value = 0.193, Egger’s *p-*value = 0.192). (EPS)

S4 Fig Funnel plot with pseudo 95% confidence limits for prospective studies of height and lung cancer (Begg’s *p-*value = 0.544, Egger’s *p-*value = 0.233). (EPS)

S5 Fig Detailed flow diagram of included studies. (TIF)

S1 Table Summary of prospective studies of height and lung cancer (*n* = 11). (DOCX)

S2 Table Summary of prospective studies of height and colorectal cancer (*n* = 24). (DOCX)

S3 Table Summary of prospective studies of height and prostate cancer (*n* = 27). (DOCX)

S4 Table Summary of prospective studies of height and aggressive prostate cancer (*n* = 12). (DOCX)

S5 Table Summary statistics for the 423 uncorrelated height-associated genetic variants. (DOCX)

S6 Table Random-effects summary estimate from published prospective studies of populations of European descent for the association between height (standardized to 10-cm increase) and multiple cancers. (DOCX)

S7 Table Inverse-variance-weighted odds ratios and 95% CIs estimated using a fixed-effects meta-analysis model for the association between adult height and multiple cancers using Mendelian randomization stratified by sex for colorectal and lung cancers. (DOCX)

S8 Table Inverse-variance-weighted odds ratios and 95% CIs estimated using a fixed-effects meta-analysis model for the association between adult height and multiple cancers using Mendelian randomization analysis with updated GWAS data excluding SNPs with demonstrated pleiotropic effects. (DOCX)

S9 Table Egger regression to assess the potential influence of pleiotropic SNPs on the Mendelian randomization summary estimate. (DOCX)

S1 Text PRISMA checklist. (DOC)

## Abbreviations

CORECT: Colorectal Transdisciplinary Study
DRIVE: Discovery, Biology, and Risk of Inherited Variants in Breast Cancer
ELLIPSE: Elucidating Loci Involved in Prostate Cancer Susceptibility
GAME-ON: Genetic Associations and Mechanisms in Oncology
GIANT: Genetic Investigation of Anthropometric Traits
GWAS: genome-wide association study
IVW: inverse-variance-weighted
OR: odds ratio
RR: relative risk
TRICL: Transdisciplinary Research in Cancer of the Lung
