# Supplementary material for: Association between Adult Height and Risk of Colorectal, Lung, and Prostate Cancer: Results from Meta-analyses of Prospective Studies and Mendelian Randomization Analyses
Source: PLoS Med. 2016 Sep 6;13(9):e1002118. doi: 10.1371/journal.pmed.1002118 (PMC5012582; doi:10.1371/journal.pmed.1002118)
Supplement: S1 Table — (DOCX) [file pmed.1002118.s006.docx]

**S1 Table**. Summary of prospective studies of height and lung cancer (n=11)

| **Author** | **Year** | **Cohort/**  **Population** | **Sex** | **Events** | **Height**  **comparison** | **Adjusted**  **Estimate(s) & CI** | **Derivation of**  **Continuous Estimate^1^** | |
| --- | --- | --- | --- | --- | --- | --- | --- | --- |
|  |  |  |  |  |  |  | **Score^2^** | **10 cm Estimate**  **& 95% CI** |
| Albanes, et al. | 1988 | NHANES | M | 114 | ≤169 cm  169.1-173.8  173.9-178.6  >178.6 | 1.0 (ref)  1.4 (0.8, 2.3)  1.6 (0.9, 2.3)  1.1 (0.6, 2.0) | 164.8  171.6  176.1  182.9 | 1.12 (0.83, 1.53) |
| Drinkard, et al. | 1995 | Iowa Women’s  Health Study | F | 233 | <155 cm (ref)  155-160  161-165  >165 | 1.00 (ref)  0.95 (0.68, 1.31)  0.87 (0.56, 1.36)  0.81 (0.57, 1.14) | 155  157.5  163  169 | 0.87 (0.70, 1.08) |
| Hebert, et al. | 1997 | Physicians’  Health  Study | M | 170 | ≤67 in  68-69  70-71  72  >73 | 1.00 (ref)  1.01 (0.63, 1.64)  0.79 (0.49, 1.27)  1.22 (0.72, 2.07)  1.07 (0.63, 1.83) | 170.18  173.99  179.07  182.88  187.96 | 1.06 (0.82, 1.38) |
| Gunnell, et al. | 2003 | U.K. South Wales | M | 78 | 6 cm increase | 1.21 (0.96, 1.51) | NA | 1.37 (0.94, 2.00) |
| Sung, et al. | 2009 | Korean | M/F | M:4453  F:943 | 5 cm increase | M: 1.07 (1.04, 1.10)  F: 1.05 (0.99, 1.13) | NA | M: 1.14 (1.08, 1.21)  F: 1.10 (0.97, 1.26) |
| Green, et al. | 2011 | U.K. Million  Women Study | F | 8,074 | 10 cm increase | 1.03 (0.98, 1.08)  Note: 99% CI | NA | 1.03 (0.99, 1.07) |
| Kabat, et al. | 2012 | Canadian National  Breast Screening  Study | F | 757 | 10 cm increase | ES: 0.93 (0.82, 1.06)  NS: 1.07 (0.78, 1.47) | NA | NA |
| Tang, et al. | 2012 | Shanghai Men’s  & Women’s  Health Study | M/F | 863 | 6 cm increase | M: 1.11 (1.00, 1.25)  F: 1.08 (0.97, 1.20) | NA | M: 1.19 (0.99, 1.43)  F: 1.14 (0.95, 1.36) |
| Kabat, et al. | 2013 | Women’s Health  Initiative | F | 1,735 | 10 cm increase | ES: 1.09 (1.00, 1.19)  NS: 1.12 (0.92, 1.38) | NA | NA |
| Walter, et al. | 2013 | VITAL | M/F | 743 | 5 in increase | 1.04 (0.90, 1.19) | NA | 1.03 (0.92, 1.16) |
| Kabat, et al. | 2014 | US NIH-AARP Diet and Health Study | M/F | M/F(NS): 230/284  M/F (ES):  5800/3201 | 10 cm increase | NS-  M: 1.18 (0.99, 1.42)  F: 1.14 (0.97, 1.35) ES-  M: 1.04 (1.00, 1.07)  F: 0.97 (0.92, 1.02) | NA | NA |

Note: M = males, F = females, ES = ever smoker, NS = never smoker, NA = not applicable

^1^For studies reporting categorical data, estimates derived using Greenland and Longnecker.

^2^Score is equivalent to the mean height value (cm) for each category, if presented in the original paper. Otherwise midrange scores were used. When using midrange scores, the score for the highest interval was determined using method presented in Il’yasova et al. where score for the uppermost open-ended category = b_n_ + (b_n_ – b_n-1_), where b_n_ represents the lower bound of the *i*th interval (*i*=1,…,n).
