# Supplementary material for: Association between Adult Height and Risk of Colorectal, Lung, and Prostate Cancer: Results from Meta-analyses of Prospective Studies and Mendelian Randomization Analyses
Source: PLoS Med. 2016 Sep 6;13(9):e1002118. doi: 10.1371/journal.pmed.1002118 (PMC5012582; doi:10.1371/journal.pmed.1002118)
Supplement: S2 Table — (DOCX) [file pmed.1002118.s007.docx]

| **Author** | **Year** | **Cohort/**  **Population** | **Sex** | **Events** | **Height**  **comparison** | **Adjusted**  **Estimate(s) & CI** | **Derivation of**  **Continuous Estimate^1^** | |
| --- | --- | --- | --- | --- | --- | --- | --- | --- |
|  |  |  |  |  |  |  | **Score^2^** | **10 cm Estimate**  **& 95% CI** |
| Suadicani, et al. | 1993 | Copenhagen | M | Rectal: 42 | 151-171 cm  172-177  178-198 (ref) | 3.1 (1.0, 9.0)  2.0 (0.7, 5.7)  1.0 (ref) | 161  174.5  188 | 0.89 (0.65, 1.21) |
| Hebert, et al. | 1997 | Physician’s  Health  Study | M | CRC: 341 | ≤67 in  68-69  70-71  72  >73 | 1.00 (ref)  1.51 (1.06, 2.14)  1.14 (0.80, 1.62)  1.19 (0.79, 1.80)  1.53 (1.04, 2.25) | 170.18  173.99  179.07  182.88  187.96 | 0.87 (0.70, 1.08) |
| Gunnell, et al. | 2003 | U.K. South Wales | M | CRC: 38 | 6 cm increase | 0.96 (0.69, 1.34) | NA | 0.93 (0.54,1.62) |
| Shimizu, et al. | 2003 | Japan | M/F | Colon: 198  M/F: 108/93  Rectal: 97  M/F: 59/41 | M/F:  ≤162/ ≤150  163-167/ 151-154  ≥168/ ≥155 | M: Colon/Rectal  1.00 (ref)  1.75(1.07,2.85)/1.87(1.02,3.44)  2.13(1.26,3.58)/1.21(0.57,2.61)  F: Colon/Rectal  1.00 (ref)  1.56(0.87,2.81)/1.27(0.53,3.06)  1.48(0.81,2.70)/1.30(0.52,3.21) | M/F:  157.5/ 146.0  164.9/ 152.6  171.7/ 158.3 | Colon:  M:1.68(1.16,2.42)  F:1.38(0.85,2.25)  Rectal:  M:1.21(0.71,2.06)  F:1.25(0.60,2.61) |
| MacInnis, et al. | 2004 | Melbourne  Collaborative  Cohort  Study | M | Colon: 153 | 10 cm increase | 1.43 (1.12, 1.83) | NA | NA |
| Wei, et al. | 2004 | Nurses’ Health  Study &  Health  Professionals  Follow-up  Study | M/F | Colon: M/F  467/672  Rectal: M/F  135/204 | M/F:  <68/<62 in  69-70/63-64  71-72/65-66  73-81/67-81 | M: Colon/Rectum  1.00 (ref)  1.11(0.87,1.43)/0.92(0.58,1.46)  1.28(1.00,1.65)/1.07(0.68,1.71)  1.50(1.13,2.00)/1.42(0.85,2.35)  F: Colon/Rectum  1.00 (ref)  1.18(0.94,1.48)/1.31(0.89,1.92)  1.22(0.97,1.52)/1.05(0.70,1.58)  1.48(1.18,1.88)/1.08(0.70,1.69) | M/F:  172.72/157.48  176.53/161.29  181.61/166.37  195.58/187.96 | Colon:  M:1.18(1.06,1.33)  F:1.11(1.04,1.19)  Rectal:  M:1.20(0.98,1.47)  F:0.98(0.86,1.12) |
| Engeland, et al. | 2005 | Norway | M/F | CRC: M/F  22,987/  24,130 | M/F:  <160 cm/ <150  160-169/ 150-159  170-179/ 160-169  180+/ 170+ | M/F:  0.86(0.74,0.99)/0.77(0.69,0.85)  0.90(0.87,0.93)/0.90(0.87,0.92)  1.00 (ref) /1.00 (ref)  1.14(1.10,1.18)/1.14(1.09,1.19) | M/F:  160/150  164.5/154.5  174.5/164.5  190/180 | M:  1.10 (1.08,1.11)  F:  1.10 (1.09,1.12) |
| Otani, et al. | 2005 | Japan | M/F:  626/360 | CRC | M/F:  <160/ <148 cm  160-162/148-150  163-165/151-153  166-169/154-156  170+/ 157+ | M/F:  1.00 (ref) / 1.00 (ref)  1.1 (0.9,1.5) / 1.3 (0.9,1.7)  1.1 (0.9,1.4) / 1.4 (0.99,1.9)  1.2 (0.9,1.6) / 1.0 (0.7,1.5)  1.1 (0.8,1.5) / 1.1 (0.7,1.6) | M/F:  160/148  161/149  164/152  167.5/155  174/160 | M:  1.05 (0.87,1.28)  F:  0.90 (0.68,1.20) |
| Bowers, et al. | 2006 | Finland | M  Smokers | CRC: 410 | 136-168cm (ref)  169-171  172-175  176-178  179-200 | CRC:  1.00 (ref)  0.87 (0.62,1.22)  1.07 (0.80,1.44)  1.23 (0.89,1.70)  0.90 (0.64,1.27) | 152  170  173.5  177  179.5 | 1.02 (0.92, 1.14) |
| MacInnis, et al. | 2006 | Melbourne  Collaborative  Cohort  Study | M/F | Rectal  M/F: 134/95 | 10 cm increase | M: 1.15 (0.89,1.48)  F: 1.38 (1.00,1.90) | NA | NA |
| MacInnis, et al. | 2006 | Melbourne  Collaborative  Cohort  Study | F | Colon: 212 | 10 cm increase | 1.17 (0.93, 1.48) | NA | NA |
| Pischon, et al. | 2006 | EPIC | M/F | Colon: M/F  421/563  Rectal: M/F  295/291 | M/F:  <168(ref) / <156  168-172.4 /156-159.9  172.5-176.1/160-163.2  176.2-180.4 /163.3-167.4  180.5+ / 167.5+ | Colon: M/F  1  1.10(0.80,1.52)/1.33(0.99,1.80)  1.16(0.84,1.60)/1.71(1.28,2.28)  1.29(0.93,1.79)/1.66(1.23,2.24)  1.40(0.99,1.98)/1.79(1.30,2.46)  Rectal: M/F  1  1.30(0.90,1.87)/1.03(0.70,1.52)  0.97(0.65,1.44)/1.25(0.86,1.81)  1.00(0.67,1.49)/0.81(0.54,1.23)  1.00(0.66,1.52)/0.78(0.50,1.21) | M/F:  168/156  170.2/157.95  174.3/161.6  178.3/165.35  184.8/171.7 | Colon:  M:1.21(1.01,1.45)  F:1.32(1.11,1.56)  Rectal:  M:0.91(0.74,1.13)  F:0.82(0.64,1.05) |
| Sung, et al. | 2009 | Korean | M/F | Colon M/F  2,499/1,007  Rectal M/F  2,281/892 | 5 cm increase | Colon M/F:  1.04(1.00,1.08)/1.08(1.01,1.15)  Rectal M/F:  1.06(1.01,1.10)/1.00(0.94,1.08) | NA | Colon:  M:1.08(1.00,1.17)  F:1.17(1.02,1.33)  Rectal:  M:1.12(1.03,1.22)  F:1.00(0.87,1.15) |
| Oxentenko, et al. | 2010 | Iowa Women’s  Health Study | F | CRC:  1,464 | ≤157 cm  158-163  164-168  169+ | 1  1.12 (0.97, 1.30)  1.22 (1.05, 1.42)  1.38 (1.17, 1.64) | 157  160.5  166  174 | 1.20 (1.09,1.31) |
| Green, et al. | 2011 | U.K. Million  Women Study | F | Colon:  6281  Rectal: 3190 | 10 cm increase | Colon: 1.25 (1.17, 1.32)  Rectal: 1.14 (1.05, 1.24)  Note: 99% CI | NA | Colon: 1.25 (1.19,1.31)  Rectal: 1.14 (1.07,1.21) |
| Hughes, et al. | 2011 | Netherlands  Cohort Study | M/F | CRC: M/F  1,211/ 1,106 | 5 cm increase | M: 0.96 (0.89, 1.04)  F: 1.09 (1.01, 1.04) | NA | M:0.92(0.79,1.08)  F:1.19(1.15,1.22) |
| Shin, et al. | 2011 | Korean | M/F | M/F:  Proximal  536/236  Distal  751/225  Rectal  1535/551 | M/F:  ≤165/ ≤151  165.1-168 /151.1-155  168.1-172 /155.1-159  >172 / >159 | Proximal M/F:  1.0(ref) / 1.0(ref)  1.0 (0.8,1.3) / 1.2 (0.9,1.7)  1.1 (0.8,1.3) / 1.3 (0.9,1.9)  1.1 (0.8,1.4) / 1.2 (0.8,1.9)  Distal M/F:  1.0(ref) / 1.0(ref)  1.3 (1.1,1.5) / 1.4 (1.0,1.9)  1.4 (1.1,1.7) / 1.5 (1.0,2.2)  1.3 (1.1,1.6) / 1.3 (0.9,2.1)  Rectal M/F:  1.0(ref)/ 1.0(ref)  1.2 (1.1,1.4) / 1.4 (1.1,1.7)  1.2 (1.0,1.4) / 1.3 (1.0,1.7)  1.1 (1.0,1.3) / 1.5 (1.1,2.0) | 165/151  166.55/153.05  170.05/157.05  175.9/162.9 | Proximal:  M:1.11(0.87,1.41)  F:1.13(0.81,1.58)  Distal:  M:1.15(0.99,1.36)  F:1.13(0.81,1.57)  Rectal:  M:1.03(0.92,1.15)  F:1.23(0.98,1.55) |
| Kabat, et al. | 2012 | Canadian National  Breast Screening  Study | F | CRC:  1096 | 10 cm increase | 1.13 (1.02, 1.24) | NA | NA |
| Tang, et al. | 2012 | Shanghai Men’s  & Women’s  Health Study | M/F | Colon M/F:  177/378  Rectum M/F:  132/239 | 6 cm increase | Colon M/F:  0.88(0.74,1.04)/1.06(0.95, 1.19)  Rectal M/F:  1.23(1.01,1.49)/0.90(0.78,1.02) | NA | Colon:  M:0.81(0.61,1.07)  F:1.10(0.91,1.33)  Rectal:  M:1.41(1.02,1.95)  F:0.84(0.67,1.05) |
| Kabat, et al. | 2013 | Women’s Health  Initiative | F | 1,904 | 10 cm increase | 1.17 (1.09, 1.26) | NA | NA |
| Walter, et al. | 2013 | VITAL | M/F | CRC: 491 | 5 in increase | 1.12 (0.94, 1.32) | NA | 1.10 (0.95,1.27) |
| Wiren, et al. | 2014 | Austria, Norway, Sweden | M/F | Colon M/F:  1634/1330  Rectum/Anus M/F:  1068/688 | 5 cm increase | Colon M/F:  1.09(1.05,1.13)/1.11(1.06,1.16)  Rectum,Anus M/F:  1.06(1.01,1.11)/1.09(1.02,1.17) | NA | Colon:  M:1.19(1.10,1.28)  F:1.23(1.13,1.35)  Rectal:  M:1.12(1.02,1.23)  F: 1.19(1.04,1.36) |
| Boursi, et al. | 2014 | UK Health Improvement Network | M/F | CRC M/F:  5617/4361 | 10 cm increase | M: 1.10 (1.05, 1.15)  F: 1.16 (1.10, 1.23) | NA | NA |
| Kabat, et al. | 2014 | US NIH-AARP Diet and Health Study | M/F | Colon M/F:  2860/1311  Rectum M/F:  1427/591 | 10 cm increase | Colon M/F:  1.10 (1.05, 1.16)/1.19 (1.10, 1.29)  Rectum M/F:  1.09 (1.02, 1.17)/1.12 (0.99, 1.26) | NA | NA |

Note: M = males, F = females, ES = ever smoker, NS = never smoker, NA = not applicable

^1^For studies reporting categorical data, estimates derived using Greenland and Longnecker.

^2^Score is equivalent to the mean height value (cm) for each category, if presented in the original paper. Otherwise midrange scores were used. When using midrange scores, the score for the highest interval was determined using method presented in Il’yasova et al. where score for the uppermost open-ended category = b_n_ + (b_n_ – b_n-1_), where b_n_ represents the lower bound of the *i*th interval (*i*=1,…,n).
