# Supplementary material for: Association between Adult Height and Risk of Colorectal, Lung, and Prostate Cancer: Results from Meta-analyses of Prospective Studies and Mendelian Randomization Analyses
Source: PLoS Med. 2016 Sep 6;13(9):e1002118. doi: 10.1371/journal.pmed.1002118 (PMC5012582; doi:10.1371/journal.pmed.1002118)
Supplement: S3 Table — (DOCX) [file pmed.1002118.s008.docx]

| **Author** | **Year** | **Cohort/**  **Population** | **Events** | **Height**  **comparison** | **Adjusted**  **Estimate(s) & CI** | **Derivation of**  **Continuous Estimate^1^** | |
| --- | --- | --- | --- | --- | --- | --- | --- |
|  |  |  |  |  |  | **Score^2^** | **10 cm Estimate**  **& 95% CI** |
| Severson, et al. | 1988 | Japanese men  (living in Hawaii) | 174 | 0-85 cm (ref)  86-87  ≥88 | 1.00 (ref)  0.97 (0.67, 1.41)  0.94 (0.66, 1.34) | 85  86.5  90 | 0.89 (0.44, 1.78) |
| Le Marchand, et al. | 1994 | Hawaii | 198 | ?  162 cm  173 cm  ? | 1.0 (ref)  1.1 (0.6, 1.8)  1.8 (1.2, 2.9)  1.8 (1.0, 3.2) | 161  162  173  174 | 1.58 (1.18, 2.11) |
| Hebert, et al. | 1997 | Physician’s Health Study | 1047 | ≤67 in  68-69  70-71  72  >73 | 1.00 (ref)  1.23 (1.00, 1.51)  1.26 (1.04, 1.54)  1.59 (1.27, 1.98)  1.26 (1.00, 1.59) | 170.18  173.99  179.07  182.88  187.96 | 1.15 (1.03, 1.29) |
| Tulinius, et al. | 1997 | Iceland | 524 | 1 cm increase | 1.01 (1.00, 1.03) | NA | 1.10 (0.95, 1.28) |
| Lund Nilsen, et al. | 1999 | Norway | 642 | ≤169 cm (ref)  170-173  174-176  177-180  ≥181 | 1.0 (ref)  1.1 (0.9, 1.4)  1.1 (0.8, 1.4)  1.2 (0.9, 1.5)  1.2 (0.9, 1.6) | 168  171.5  175  178.5  185 | 1.11 (0.95, 1.29) |
| Habel, et al. | 2000 | Kaiser Permenente  Cohort | 2079 | <66.6 in (ref)  66.6-68.1  68.2-69.6  69.7-71.2  >71.2 | 1.00 (ref)  1.10 (0.97, 1.26)  1.03 (0.90, 1.18)  1.16 (1.01, 1.33)  1.15 (1.00, 1.33) | 168.91  171.07  175.01  178.94  184.66 | 1.06 (1.00, 1.12) |
| Putnam, et al. | 2000 | Iowa | 101 | <175 cm (ref)  175-179  >180 | 1.00 (ref)  0.9 (0.5, 1.5) 1.1 (0.7, 1.7) | 174  177  185 | 1.11 (1.06, 1.17) |
| Schuurman, et al. | 2000 | Netherlands Cohort  Study | 681 | 5 cm increase | 0.99 (0.92, 1.06) | NA | 0.98 (0.85, 1.13) |
| Engeland, et al. | 2003 | Norway | 33300 | < 160 cm  160-169  170-179 (ref)  180-189  ≥ 190 | 0.64 (0.57, 0.73)  0.89 (0.87, 0.92)  1.00 (ref)  1.06 (1.03, 1.09)  1.11 (0.99, 1.24) | 159  164.5  174.5  184.5  200 | 1.10 (1.08, 1.11) |
| Gunnell, et al. | 2003 | U.K. South Wales | 33 | 6 cm increase | 0.88 (0.61, 1.20) | NA | 0.81 (0.46, 1.42) |
| Gong, et al. | 2006 | Prostate Cancer  Prevention Trial | 1936 | <172 cm (ref)  172-178  179-182  ≥183 | 1.00 (ref)  1.07 (0.94, 1.23)  1.07 (0.93, 1.23)  1.22 (1.05, 1.43) | 171  175  180.5  187 | 1.12 (1.02, 1.22) |
| Kurahashi, et al. | 2006 | Japan | 311 | ≤159 (ref)  160-164  164-167  ≥ 168 | 1.00 (ref)  1.27 (0.93, 1.73)  1.24 (0.88, 1.75)  1.08 (0.73, 1.59) | 159  162  165.5  172 | 0.98 (0.74, 1.29) |
| Sequoia, et al. | 2006 | Alpha-Tocopherol,  Beta-Carotene Cancer  Prevention Study cohort | 1346 | 136-168 cm  169-171  172-175  176-178  179-200 | 1.00 (ref)  1.11 (0.93, 1.32)  1.11 (0.95, 1.31)  1.30 (1.09, 1.55)  1.14 (0.96, 1.35) | 152  170  173.5  177  179.5 | 1.07 (1.01, 1.13) |
| Giovannucci, et al. | 2007 | Health Professionals  Follow-up Study | 3544 | <66 in (ref)  >72 in  ~22.86 cm increase | 1.0 (ref)  1.05 (0.88, 1.27) | 165.1  187.96 | 1.02 (0.94, 1.11) |
| Littman, et al. | 2007 | VITAL | 832 | ≤ 68 in (ref)  69-70  71-72  ≥73 | 1.0 (ref)  1.2 (0.98, 1.5)  1.1 (0.91, 1.4)  1.3 (1.1, 1.6) | 68  69.5  71.5  75 | 1.34 (1.06, 1.71) |
| Pischon, et al. | 2008 | EPIC | 2446 | 5 cm increase | 1.01 (0.98, 1.04) | NA | 1.02 (0.96, 1.08) |
| Zuccolo, et al. | 2008 | ProtecT RCT | 1375 | 10 cm increase | 1.06 (0.97, 1.16) | NA | NA |
| Ahn, et al. | 2009 | PLCO | 2144 | 5 cm increase | 1.02 (0.98, 1.05) | NA | 1.04 (0.97, 1.11) |
| Hernandez, et al. | 2009 | Multiethnic cohort | 5554 | <66 in (ref)  66-67.9  68-69.9  ≥70 | 1.00 (ref)  0.99 (0.91, 1.08)  0.98 (0.89, 1.08)  1.01 (0.92, 1.11) | 165.1  170.05  175.13  182.88 | 1.01 (0.96, 1.06) |
| Sung, et al. | 2009 | Korean | 1612 | 5 cm increase | 1.08 (1.03, 1.13) | NA | 1.17 (1.06, 1.28) |
| Wallstrom, et al. | 2009 | Malmo Diet and Cancer study (Sweden) | 817 | ≤170 cm (ref)  171-174  175-178  179-181  ≥182 | 1.00 (ref)  1.20 (0.97, 1.49)  1.11 (0.89, 1.38)  1.09 (0.85, 1.39)  1.40 (1.13, 1.74) | 168  173  177  180  184 | 1.18 (1.04, 1.33) |
| Stocks, et al. | 2010 | Swedish Male Construction Workers | 10002 | < 173 cm (ref)  173-177  177-180  180-184  ≥184 | 1.00 (ref)  1.06 (1.01, 1.12)  1.10 (1.04, 1.17)  1.16 (1.08, 1.23)  1.14 (1.06, 1.22) | 172  175  178.5  182  188 | 1.09 (1.05, 1.13) |
| Bassett, et al. | 2012 | Melbourne Collaborative  Cohort Study | 1374 | 5 cm increase | 1.02 (0.97, 1.07) | NA | 1.04 (0.94, 1.15) |
| Shafique, et al. | 2012 | UK | 650 | ≤165.1 cm  165.2-170  170.1-172.72  172.73-177.8  ≥177.9 | 1.00 (ref)  1.05 (0.82, 1.35)  1.11 (0.86, 1.43)  1.27 (1.01, 1.61)  1.35 (1.04, 1.75) | 165.1  167.6  171.41  175.27  183.07 | 1.20 (1.05, 1.36) |
| Tang, et al. | 2012 | Shanghai Men’s Health Study | 165 | 6 cm increase | 0.78 (0.66, 0.94) | NA | 0.66 (0.49, 0.89) |
| Wiren, et al. | 2014 | Austria, Norway, Sweden | 6176 | 5 cm increase | 1.05 (1.03, 1.07) | NA | 1.10 (1.06, 1.15) |
| Kabat, et al. | 2014 | US NIH-AARP Diet and Health Study | 23097 | 10 cm increase | 1.02 (1.00, 1.04) | NA | NA |

Note: NA = not applicable

^1^For studies reporting categorical data, estimates derived using Greenland and Longnecker.

^2^Score is equivalent to the mean height value (cm) for each category, if presented in the original paper. Otherwise midrange scores were used. When using midrange scores, the score for the highest interval was determined using method presented in Il’yasova et al. where score for the uppermost open-ended category = b_n_ + (b_n_ – b_n-1_), where b_n_ represents the lower bound of the *i*th interval (*i*=1,…,n).
