# Supplementary material for: Association between Adult Height and Risk of Colorectal, Lung, and Prostate Cancer: Results from Meta-analyses of Prospective Studies and Mendelian Randomization Analyses
Source: PLoS Med. 2016 Sep 6;13(9):e1002118. doi: 10.1371/journal.pmed.1002118 (PMC5012582; doi:10.1371/journal.pmed.1002118)
Supplement: S4 Table — (DOCX) [file pmed.1002118.s009.docx]

| **Author** | **Year** | **Cohort/**  **Population** | **Events** | **Height**  **comparison** | **Adjusted**  **Estimate(s) & CI** | **Derivation of**  **Continuous Estimate^1^** | |
| --- | --- | --- | --- | --- | --- | --- | --- |
|  |  |  |  |  |  | **Score^2^** | **10 cm Estimate**  **& 95% CI** |
| Gong, et al. | 2006 | Prostate Cancer  Prevention Trial | 521 | <172 cm (ref)  172-178  179-182  ≥183 | 1.00 (ref)  1.17 (0.92, 1.47)  1.11 (0.87, 1.42)  1.18 (0.88, 1.57) | 171  175  180.5  187 | 1.07 (0.91, 1.27) |
| Kurahashi, et al. | 2006 | Japan | 91 | ≤159 (ref)  160-164  164-167  ≥ 168 | 1.00 (ref)  1.57 (0.88, 2.81)  1.31 (0.68, 2.53)  1.51 (0.74, 3.10) | 159  162  165.5  172 | 1.13 (0.70, 1.84) |
| Giovannucci, et al. | 2007 | Health Professionals  Follow-up Study | 1110 | <66 in (ref)  >72 in  ~22.86 cm increase | 1.0 (ref)  1.07 (0.77, 1.48) | 165.1  187.96 | 1.03 (0.89, 1.19) |
| Littman, et al. | 2007 | VITAL | 378 | ≤ 68 in (ref)  69-70  71-72  ≥73 | 1.0 (ref)  1.0 (0.75, 1.4)  1.3 (0.98, 1.7)  1.4 (0.98, 1.9) | 68  69.5  71.5  75 | 1.75 (1.14, 2.69) |
| Pischon, et al. | 2008 | EPIC | 580 | 5 cm increase | 1.01 (0.95, 1.08) | NA | 1.02 (0.90, 1.16) |
| Zuccolo, et al. | 2008 | ProtecT RCT | 402 | 10 cm increase | 1.23 (1.06, 1.43) | NA | NA |
| Ahn, et al. | 2009 | PLCO | 912 | 5 cm increase | 1.05 (1.00, 1.10) | NA | 1.10 (1.00, 1.21) |
| Hernandez, et al. | 2009 | Multiethnic cohort | 1563 | <66 in (ref)  66-67.9  68-69.9  ≥70 | 1.00 (ref)  1.08 (0.92, 1.26)  1.04 (0.87, 1.24)  1.09 (0.91, 1.30) | 165.1  170.05  175.13  182.88 | 1.04 (0.94, 1.14) |
| Wallstrom, et al. | 2009 | Malmo Diet and Cancer study (Sweden) | 281 | ≤170 cm (ref)  171-174  175-178  179-181  ≥182 | 1.00 (ref)  1.40 (0.97, 2.01)  1.20 (0.83, 1.74)  1.06 (0.69, 1.63)  1.38 (0.95, 2.02) | 168  173  177  180  184 | 1.10 (0.89, 1.37) |
| Stocks, et al. | 2010 | Swedish Male Construction Workers | 2408 | < 173 cm (ref)  173-177  177-180  180-184  ≥184 | 1.00 (ref)  1.03 (0.93, 1.15)  1.02 (0.91, 1.16)  1.12 (0.98, 1.28)  0.95 (0.83, 1.09) | 172  175  178.5  182  188 | 0.98 (0.91, 1.06) |
| Bassett, et al. | 2012 | Melbourne Collaborative  Cohort Study | 410 | 5 cm increase | 0.91 (0.83, 0.99) | NA | 0.83 (0.69, 0.99) |
| Shafique, et al. | 2012 | UK | 119 | ≤165.1 cm  165.2-170  170.1-172.72  172.73-177.8  ≥177.9 | 1.00 (ref)  1.27 (0.68, 2.37)  1.54 (0.61, 2.19)  1.69 (0.94, 3.05)  1.32 (0.68, 2.55) | 165.1  167.6  171.41  175.27  183.07 | 1.17 (0.85, 1.62) |

Note: NA = not applicable

^1^For studies reporting categorical data, estimates derived using Greenland and Longnecker.

^2^Score is equivalent to the mean height value (cm) for each category, if presented in the original paper. Otherwise midrange scores were used. When using midrange scores, the score for the highest interval was determined using method presented in Il’yasova et al. where score for the uppermost open-ended category = b_n_ + (b_n_ – b_n-1_), where b_n_ represents the lower bound of the *i*th interval (*i*=1,…,n).
