# Supplementary material for: Association between Adult Height and Risk of Colorectal, Lung, and Prostate Cancer: Results from Meta-analyses of Prospective Studies and Mendelian Randomization Analyses
Source: PLoS Med. 2016 Sep 6;13(9):e1002118. doi: 10.1371/journal.pmed.1002118 (PMC5012582; doi:10.1371/journal.pmed.1002118)
Supplement: S5 Table — (DOCX) [file pmed.1002118.s010.docx]

**S5 Table.**  Summary statistics for the 423 uncorrelated height-associated genetic variants

| **No** | **Chr** | **SNP** | **GRCh36/hg18** | **Nearest**  **gene** | **Effect**  **allele** | **Ref**  **allele** | **Height GWAS**  **(from Wood, et al.)** | |  | **CORECT**  **(n=346)** | |  | **DRIVE**  **(n=423)** | |  | **ELLIPSE**  **(n=419)** | |  | **TRICL**  **(n=423)** | |
| --- | --- | --- | --- | --- | --- | --- | --- | --- | --- | --- | --- | --- | --- | --- | --- | --- | --- | --- | --- | --- |
|  |  |  |  |  |  |  | **β** | **SE** |  | **β** | **SE** |  | **β** | **SE** |  | **β** | **SE** |  | **β** | **SE** |
| 1 | 1 | rs425277 | 2,059,032 | PRKCZ | T | C | 0.0279 | 0.0030 |  | -0.0328 | 0.0568 |  | -0.0138 | 0.0192 |  | 0.0304 | 0.0236 |  | -0.0309 | 0.0193 |
| 2 | 1 | rs9434723 | 9,214,869 | H6PD | A | G | 0.0290 | 0.0040 |  | 0.1002 | 0.0723 |  | -0.0201 | 0.0250 |  | 0.0133 | 0.0298 |  | 0.0227 | 0.0239 |
| 3 | 1 | rs10779751 | 11,206,923 | FRAP1 | A | G | 0.0206 | 0.0030 |  | -0.0177 | 0.0568 |  | -0.0132 | 0.0192 |  | 0.0181 | 0.0240 |  | 0.0404 | 0.0191 |
| 4 | 1 | rs2284746 | 17,179,262 | MFAP2 | G | C | 0.0399 | 0.0030 |  | -0.0875 | 0.0518 |  | 0.0177 | 0.0176 |  | 0.0401 | 0.0192 |  | -0.0143 | 0.0271 |
| 5 | 1 | rs12137162 | 19,635,983 | CAPZB | A | C | 0.0191 | 0.0030 |  | -0.0452 | 0.0575 |  | 0.0263 | 0.0191 |  | 0.0007 | 0.0237 |  | 0.0213 | 0.0189 |
| 6 | 1 | rs212524 | 21,455,898 | ECE1 | C | T | 0.0206 | 0.0030 |  | -0.0333 | 0.0514 |  | 0.0371 | 0.0178 |  | 0.0266 | 0.0199 |  | 0.0084 | 0.0174 |
| 7 | 1 | rs2806561 | 23,377,382 | LUZP1 | A | G | 0.0272 | 0.0030 |  | 0.0551 | 0.0515 |  | -0.0168 | 0.0173 |  | 0.0028 | 0.0214 |  | -0.0164 | 0.0171 |
| 8 | 1 | rs4601530 | 24,916,698 | CLIC4 | C | T | 0.0253 | 0.0030 |  | 0.0092 | 0.0569 |  | 0.0069 | 0.0197 |  | -0.0119 | 0.0205 |  | -0.0179 | 0.0193 |
| 9 | 1 | rs16834765 | 32,144,029 | PTP4A2 | T | C | 0.0449 | 0.0060 |  | -0.0792 | 0.0994 |  | -0.0127 | 0.0373 |  | 0.0064 | 0.0462 |  | -0.0004 | 0.0367 |
| 10 | 1 | rs7544462 | 37,735,343 | C1orf149 | A | C | 0.0322 | 0.0050 |  | 0.1078 | 0.1069 |  | 0.0086 | 0.0323 |  | -0.0934 | 0.0360 |  | -0.0101 | 0.0324 |
| 11 | 1 | rs6600365 | 41,328,840 | SCMH1 | C | T | 0.0272 | 0.0030 |  | 0.0032 | 0.0512 |  | -0.0193 | 0.0172 |  | -0.0636 | 0.0186 |  | 0.0244 | 0.0170 |
| 12 | 1 | rs3014219 | 45,797,041 | AKR1A1 | G | A | 0.0210 | 0.0030 |  | . | . |  | -0.0111 | 0.0175 |  | . | . |  | -0.0321 | 0.0269 |
| 13 | 1 | rs564914 | 47,687,820 | FOXD2 | T | A | 0.0242 | 0.0030 |  | -0.0150 | 0.0522 |  | 0.0062 | 0.0181 |  | 0.0066 | 0.0219 |  | -0.0262 | 0.0178 |
| 14 | 1 | rs12855 | 51,212,681 | CDKN2C | T | C | 0.0378 | 0.0050 |  | -0.0858 | 0.0853 |  | 0.0183 | 0.0298 |  | -0.0232 | 0.0368 |  | -0.0043 | 0.0299 |
| 15 | 1 | rs6691924 | 54,726,833 | ACOT11 | T | C | 0.0318 | 0.0050 |  | . | . |  | -0.0374 | 0.0289 |  | -0.0177 | 0.0346 |  | 0.0358 | 0.0274 |
| 16 | 1 | rs2815379 | 67,283,062 | SLC35D1 | G | A | 0.0184 | 0.0030 |  | . | . |  | -0.0089 | 0.0191 |  | 0.0011 | 0.0212 |  | -0.0336 | 0.0205 |
| 17 | 1 | rs17391694 | 78,396,214 | GIPC2 | T | C | 0.0428 | 0.0050 |  | 0.0606 | 0.3007 |  | 0.0167 | 0.0293 |  | 0.0022 | 0.0312 |  | 0.1385 | 0.0313 |
| 18 | 1 | rs567401 | 85,760,746 | DDAH1 | T | C | 0.0247 | 0.0040 |  | 0.1362 | 0.0778 |  | -0.0015 | 0.0248 |  | -0.0059 | 0.0277 |  | 0.0088 | 0.0268 |
| 19 | 1 | rs7551732 | 88,911,629 | PKN2 | A | T | 0.0273 | 0.0030 |  | 0.0459 | 0.0518 |  | 0.0205 | 0.0175 |  | -0.0191 | 0.0187 |  | 0.0014 | 0.0195 |
| 20 | 1 | rs2811594 | 93,115,870 | FAM69A | G | A | 0.0241 | 0.0030 |  | -0.0483 | 0.0536 |  | -0.0107 | 0.0192 |  | -0.0306 | 0.0196 |  | -0.0492 | 0.0179 |
| 21 | 1 | rs17113369 | 95,559,811 | RWDD3 | T | C | 0.0734 | 0.0130 |  | 0.0031 | 0.1208 |  | 0.1161 | 0.0945 |  | 0.0111 | 0.0545 |  | 0.0328 | 0.0485 |
| 22 | 1 | rs7517682 | 103,292,177 | COL11A1 | G | A | 0.0226 | 0.0030 |  | -0.0442 | 0.0515 |  | 0.0060 | 0.0175 |  | -0.0240 | 0.0216 |  | -0.0062 | 0.0175 |
| 23 | 1 | rs12120956 | 113,004,094 | CAPZA1 | G | A | 0.0247 | 0.0040 |  | -0.0213 | 0.0632 |  | 0.0190 | 0.0211 |  | -0.0040 | 0.0228 |  | -0.0062 | 0.0206 |
| 24 | 1 | rs9428104 | 118,657,110 | SPAG17 | G | A | 0.0428 | 0.0030 |  | 0.0162 | 0.0573 |  | 0.0216 | 0.0202 |  | 0.0470 | 0.0249 |  | 0.0031 | 0.0211 |
| 25 | 1 | rs6658763 | 145,158,997 | FMO5 | C | T | 0.0359 | 0.0050 |  | 0.0729 | 0.0913 |  | -0.0261 | 0.0316 |  | -0.0271 | 0.0396 |  | 0.0252 | 0.0312 |
| 26 | 1 | rs7534365 | 148,142,748 | SV2A | C | T | 0.0473 | 0.0050 |  | . | . |  | 0.0700 | 0.0323 |  | -0.0157 | 0.0363 |  | -0.0012 | 0.0322 |
| 27 | 1 | rs2298265 | 149,525,667 | ZNF687 | C | T | 0.0299 | 0.0050 |  | -0.0794 | 0.0762 |  | 0.0286 | 0.0273 |  | 0.0089 | 0.0313 |  | -0.0154 | 0.0261 |
| 28 | 1 | rs6688100 | 158,666,210 | VANGL2 | T | C | 0.0164 | 0.0030 |  | 0.0700 | 0.0506 |  | -0.0102 | 0.0174 |  | 0.0326 | 0.0215 |  | 0.0261 | 0.0169 |
| 29 | 1 | rs4656220 | 168,915,901 | PRRX1 | T | C | 0.0206 | 0.0030 |  | . | . |  | -0.0199 | 0.0194 |  | 0.0021 | 0.0224 |  | 0.0187 | 0.0178 |
| 30 | 1 | rs6694089 | 170,350,504 | DNM3 | A | G | 0.0389 | 0.0030 |  | 0.0291 | 0.0567 |  | 0.0163 | 0.0192 |  | -0.0125 | 0.0226 |  | 0.0082 | 0.0189 |
| 31 | 1 | rs1325596 | 175,060,689 | PAPPA2 | A | G | 0.0252 | 0.0030 |  | . | . |  | -0.0331 | 0.0173 |  | -0.0038 | 0.0196 |  | 0.0449 | 0.0171 |
| 32 | 1 | rs3814333 | 182,273,742 | GLT25D2 | T | C | 0.0486 | 0.0030 |  | -0.1294 | 0.0564 |  | 0.0411 | 0.0191 |  | -0.0131 | 0.0234 |  | -0.0067 | 0.0188 |
| 33 | 1 | rs2275325 | 202,067,358 | ZC3H11A | C | G | 0.0194 | 0.0030 |  | 0.0159 | 0.0580 |  | 0.0532 | 0.0194 |  | -0.0251 | 0.0223 |  | 0.0037 | 0.0190 |
| 34 | 1 | rs10863936 | 210,304,421 | DTL | G | A | 0.0200 | 0.0030 |  | 0.0074 | 0.0514 |  | 0.0002 | 0.0173 |  | 0.0027 | 0.0198 |  | 0.0109 | 0.0170 |
| 35 | 1 | rs6540834 | 212,694,042 | PTPN14 | C | T | 0.0272 | 0.0030 |  | 0.0553 | 0.0518 |  | 0.0038 | 0.0195 |  | 0.0084 | 0.0204 |  | -0.0003 | 0.0182 |
| 36 | 1 | rs991967 | 216,682,074 | TGFB2 | C | A | 0.0344 | 0.0030 |  | 0.0784 | 0.0564 |  | 0.0097 | 0.0190 |  | -0.0274 | 0.0221 |  | 0.0114 | 0.0187 |
| 37 | 1 | rs1935157 | 219,383,881 | HLX | G | C | 0.0248 | 0.0030 |  | 0.0706 | 0.0560 |  | -0.0028 | 0.0190 |  | -0.0167 | 0.0208 |  | -0.0249 | 0.0189 |
| 38 | 1 | rs1544196 | 222,699,405 | WDR26 | G | A | 0.0186 | 0.0040 |  | -0.0044 | 0.0626 |  | -0.0011 | 0.0205 |  | 0.0018 | 0.0236 |  | -0.0151 | 0.0202 |
| 39 | 1 | rs6696239 | 225,816,691 | ZNF678 | G | A | 0.0376 | 0.0040 |  | -0.0983 | 0.0676 |  | 0.0386 | 0.0225 |  | 0.0083 | 0.0236 |  | -0.0138 | 0.0245 |
| 40 | 1 | rs11799609 | 241,684,940 | SDCCAG8 | T | G | 0.0259 | 0.0040 |  | 0.1850 | 0.0739 |  | 0.0142 | 0.0244 |  | -0.0058 | 0.0247 |  | 0.0458 | 0.0230 |
| 41 | 2 | rs17038954 | 1,624,680 | PXDN | T | C | 0.0443 | 0.0060 |  | 0.0302 | 0.1108 |  | -0.0040 | 0.0375 |  | 0.0153 | 0.0406 |  | -0.0621 | 0.0401 |
| 42 | 2 | rs3885668 | 10,095,930 | KLF11 | C | T | 0.0217 | 0.0030 |  | 0.0232 | 0.0518 |  | 0.0079 | 0.0182 |  | 0.0141 | 0.0202 |  | -0.0197 | 0.0174 |
| 43 | 2 | rs2345835 | 18,438,433 | RDH14 | C | T | 0.0181 | 0.0030 |  | -0.0341 | 0.0507 |  | -0.0095 | 0.0177 |  | 0.0082 | 0.0202 |  | 0.0295 | 0.0171 |
| 44 | 2 | rs13006748 | 20,015,300 | WDR35 | C | G | 0.0225 | 0.0030 |  | -0.1067 | 0.0567 |  | 0.0206 | 0.0205 |  | 0.0182 | 0.0221 |  | -0.0014 | 0.0194 |
| 45 | 2 | rs2289195 | 25,316,987 | DNMT3A | A | G | 0.0382 | 0.0030 |  | . | . |  | 0.0425 | 0.0176 |  | -0.0239 | 0.0217 |  | -0.0146 | 0.0175 |
| 46 | 2 | rs780094 | 27,594,741 | GCKR | C | T | 0.0206 | 0.0030 |  | 0.0260 | 0.0520 |  | 0.0511 | 0.0177 |  | -0.0137 | 0.0183 |  | -0.0014 | 0.0174 |
| 47 | 2 | rs6714546 | 33,214,929 | LTBP1 | G | A | 0.0301 | 0.0030 |  | -0.0036 | 0.0574 |  | 0.0076 | 0.0207 |  | 0.0356 | 0.0220 |  | 0.0216 | 0.0192 |
| 48 | 2 | rs17511102 | 37,814,117 | CDC42EP3 | T | A | 0.0528 | 0.0060 |  | -0.0782 | 0.0991 |  | 0.0134 | 0.0332 |  | -0.0032 | 0.0431 |  | 0.0236 | 0.0369 |
| 49 | 2 | rs13416119 | 42,316,434 | EML4 | A | G | 0.0285 | 0.0050 |  | 0.0419 | 0.0938 |  | 0.0406 | 0.0295 |  | -0.0128 | 0.0345 |  | -0.0142 | 0.0288 |
| 50 | 2 | rs9309101 | 43,483,116 | THADA | G | A | 0.0206 | 0.0030 |  | -0.0355 | 0.0535 |  | 0.0267 | 0.0183 |  | -0.0009 | 0.0189 |  | 0.0174 | 0.0179 |
| 51 | 2 | rs897080 | 44,627,706 | C2orf34 | C | T | 0.0282 | 0.0030 |  | . | . |  | 0.0422 | 0.0204 |  | 0.0380 | 0.0230 |  | -0.0028 | 0.0202 |
| 52 | 2 | rs12474201 | 46,774,789 | SOCS5 | A | G | 0.0277 | 0.0030 |  | 0.0547 | 0.0530 |  | -0.0014 | 0.0184 |  | -0.0055 | 0.0194 |  | -0.0368 | 0.0182 |
| 53 | 2 | rs354196 | 54,819,911 | SPTBN1 | G | A | 0.0209 | 0.0030 |  | 0.0015 | 0.0518 |  | 0.0029 | 0.0177 |  | -0.0001 | 0.0197 |  | 0.0084 | 0.0173 |
| 54 | 2 | rs3791679 | 55,950,396 | EFEMP1 | A | G | 0.0600 | 0.0040 |  | 0.0050 | 0.0592 |  | 0.0140 | 0.0203 |  | 0.0123 | 0.0232 |  | 0.0289 | 0.0200 |
| 55 | 2 | rs2120335 | 68,348,506 | PPP3R1 | G | A | 0.0186 | 0.0030 |  | -0.0284 | 0.0525 |  | 0.0108 | 0.0189 |  | -0.0024 | 0.0289 |  | 0.0026 | 0.0186 |
| 56 | 2 | rs7568069 | 71,437,993 | ZNF638 | G | A | 0.0216 | 0.0030 |  | -0.0301 | 0.0513 |  | 0.0020 | 0.0176 |  | -0.0329 | 0.0195 |  | 0.0069 | 0.0171 |
| 57 | 2 | rs11684404 | 88,705,737 | EIF2AK3 | C | T | 0.0316 | 0.0030 |  | 0.0938 | 0.0546 |  | 0.0482 | 0.0183 |  | 0.0150 | 0.0192 |  | 0.0199 | 0.0182 |
| 58 | 2 | rs11683207 | 97,699,722 | ZAP70 | T | C | 0.0228 | 0.0040 |  | . | . |  | 0.0041 | 0.0238 |  | 0.0234 | 0.0343 |  | -0.0534 | 0.0240 |
| 59 | 2 | rs13388725 | 108,413,622 | GCC2 | G | A | 0.0179 | 0.0030 |  | -0.0880 | 0.0520 |  | -0.0439 | 0.0179 |  | 0.0044 | 0.0204 |  | -0.0137 | 0.0176 |
| 60 | 2 | rs2166898 | 121,329,129 | GLI2 | G | A | 0.0266 | 0.0040 |  | -0.0436 | 0.0694 |  | -0.0472 | 0.0246 |  | 0.0430 | 0.0289 |  | -0.0265 | 0.0234 |
| 61 | 2 | rs7567288 | 134,151,294 | NAP5 | C | T | 0.0285 | 0.0040 |  | 0.0197 | 0.0669 |  | 0.0096 | 0.0230 |  | 0.0043 | 0.0271 |  | 0.0496 | 0.0223 |
| 62 | 2 | rs4953951 | 135,903,815 | ZRANB3 | C | T | 0.0360 | 0.0050 |  | -0.0807 | 0.0783 |  | 0.0408 | 0.0286 |  | 0.0268 | 0.0383 |  | 0.0084 | 0.0278 |
| 63 | 2 | rs749234 | 144,947,819 | ZEB2 | A | G | 0.0174 | 0.0030 |  | 0.0831 | 0.0549 |  | 0.0458 | 0.0188 |  | -0.0542 | 0.0230 |  | 0.0068 | 0.0184 |
| 64 | 2 | rs540652 | 169,415,674 | NOSTRIN | T | C | 0.0206 | 0.0030 |  | 0.0975 | 0.0512 |  | 0.0084 | 0.0175 |  | 0.0084 | 0.0200 |  | 0.0045 | 0.0172 |
| 65 | 2 | rs12987566 | 171,860,892 | METTL8 | T | C | 0.0235 | 0.0030 |  | . | . |  | 0.0285 | 0.0196 |  | 0.0343 | 0.0223 |  | 0.0168 | 0.0195 |
| 66 | 2 | rs6746356 | 174,524,144 | SP3 | A | C | 0.0193 | 0.0030 |  | -0.0217 | 0.0597 |  | -0.0139 | 0.0199 |  | -0.0057 | 0.0222 |  | -0.0023 | 0.0201 |
| 67 | 2 | rs7567851 | 178,392,966 | PDE11A | C | G | 0.0394 | 0.0060 |  | 0.2097 | 0.0990 |  | 0.0116 | 0.0308 |  | 0.0073 | 0.0338 |  | -0.0050 | 0.0315 |
| 68 | 2 | rs833152 | 182,927,346 | PDE1A | C | A | 0.0166 | 0.0030 |  | 0.0289 | 0.0523 |  | 0.0069 | 0.0176 |  | -0.0222 | 0.0196 |  | -0.0281 | 0.0173 |
| 69 | 2 | rs12693589 | 191,540,907 | STAT1 | C | T | 0.0221 | 0.0030 |  | -0.0147 | 0.0584 |  | 0.0531 | 0.0200 |  | 0.0139 | 0.0249 |  | 0.0149 | 0.0218 |
| 70 | 2 | rs6435143 | 202,902,501 | NOP5/NOP58 | A | C | 0.0190 | 0.0030 |  | 0.0056 | 0.0519 |  | -0.0453 | 0.0174 |  | -0.0123 | 0.0205 |  | -0.0121 | 0.0174 |
| 71 | 2 | rs4425077 | 216,118,761 | FN1 | G | C | 0.0195 | 0.0030 |  | -0.0621 | 0.0522 |  | 0.0331 | 0.0184 |  | -0.0281 | 0.0199 |  | 0.0484 | 0.0282 |
| 72 | 2 | rs12470505 | 219,616,613 | CCDC108 | T | G | 0.0475 | 0.0050 |  | . | . |  | 0.0341 | 0.0292 |  | -0.0063 | 0.0369 |  | 0.0052 | 0.0287 |
| 73 | 2 | rs12621643 | 223,626,227 | KCNE4 | G | T | 0.0166 | 0.0030 |  | 0.0477 | 0.0548 |  | -0.0121 | 0.0205 |  | -0.0009 | 0.0210 |  | -0.0144 | 0.0200 |
| 74 | 2 | rs6761041 | 224,738,373 | SERPINE2 | T | C | 0.0233 | 0.0030 |  | -0.0029 | 0.0511 |  | 0.0055 | 0.0182 |  | 0.0342 | 0.0197 |  | 0.0043 | 0.0173 |
| 75 | 2 | rs3116168 | 232,698,075 | DIS3L2 | C | T | 0.0388 | 0.0030 |  | 0.0336 | 0.0554 |  | 0.0260 | 0.0193 |  | -0.0376 | 0.0216 |  | 0.0198 | 0.0189 |
| 76 | 2 | rs11687941 | 241,840,083 | HDLBP | C | G | 0.0250 | 0.0030 |  | 0.0733 | 0.0581 |  | 0.0174 | 0.0199 |  | 0.0103 | 0.0224 |  | 0.0067 | 0.0198 |
| 77 | 3 | rs2633761 | 4,703,104 | ITPR1 | A | G | 0.0164 | 0.0030 |  | . | . |  | 0.0368 | 0.0173 |  | 0.0244 | 0.0194 |  | 0.0109 | 0.0171 |
| 78 | 3 | rs2597513 | 13,530,836 | HDAC11 | C | T | 0.0394 | 0.0050 |  | 0.0495 | 0.0814 |  | -0.0025 | 0.0283 |  | -0.0003 | 0.0346 |  | -0.0107 | 0.0277 |
| 79 | 3 | rs9816693 | 38,022,958 | VILL | C | G | 0.0314 | 0.0040 |  | -0.0427 | 0.0686 |  | -0.0237 | 0.0239 |  | 0.0363 | 0.0262 |  | 0.0026 | 0.0232 |
| 80 | 3 | rs3915129 | 41,218,746 | CTNNB1 | G | T | 0.0160 | 0.0030 |  | 0.0904 | 0.0509 |  | 0.0065 | 0.0174 |  | 0.0028 | 0.0214 |  | 0.0115 | 0.0172 |
| 81 | 3 | rs13088462 | 51,046,753 | DOCK3 | C | T | 0.0586 | 0.0070 |  | -0.1553 | 0.1226 |  | 0.0948 | 0.0411 |  | 0.0749 | 0.0439 |  | -0.0028 | 0.0426 |
| 82 | 3 | rs2581830 | 53,109,138 | RFT1 | T | C | 0.0306 | 0.0030 |  | 0.0415 | 0.0522 |  | -0.0200 | 0.0175 |  | 0.0113 | 0.0184 |  | -0.0178 | 0.0172 |
| 83 | 3 | rs2034172 | 55,386,803 | WNT5A | G | A | 0.0179 | 0.0030 |  | -0.0114 | 0.0537 |  | 0.0060 | 0.0191 |  | -0.0273 | 0.0193 |  | 0.0371 | 0.0188 |
| 84 | 3 | rs9835332 | 56,642,722 | C3orf63 | G | C | 0.0282 | 0.0030 |  | . | . |  | 0.0167 | 0.0173 |  | -0.0300 | 0.0199 |  | 0.0242 | 0.0264 |
| 85 | 3 | rs1658351 | 57,988,613 | FLNB | C | T | 0.0225 | 0.0030 |  | . | . |  | -0.0014 | 0.0188 |  | -0.0056 | 0.0207 |  | 0.0001 | 0.0185 |
| 86 | 3 | rs6794009 | 61,488,535 | PTPRG | G | A | 0.0161 | 0.0030 |  | 0.0262 | 0.0516 |  | -0.0021 | 0.0174 |  | -0.0028 | 0.0191 |  | 0.0132 | 0.0171 |
| 87 | 3 | rs17806888 | 67,499,012 | SUCLG2 | T | C | 0.0338 | 0.0050 |  | 0.0093 | 0.0734 |  | -0.0031 | 0.0270 |  | 0.0398 | 0.0313 |  | -0.0050 | 0.0270 |
| 88 | 3 | rs2175513 | 68,705,056 | FAM19A1 | G | A | 0.0167 | 0.0030 |  | -0.0211 | 0.0515 |  | 0.0196 | 0.0178 |  | 0.0014 | 0.0201 |  | -0.0127 | 0.0174 |
| 89 | 3 | rs12330322 | 72,538,045 | RYBP | C | T | 0.0343 | 0.0040 |  | -0.0323 | 0.0622 |  | 0.0079 | 0.0206 |  | -0.0331 | 0.0260 |  | -0.0276 | 0.0207 |
| 90 | 3 | rs9825951 | 100,752,611 | COL8A1 | T | A | 0.0220 | 0.0030 |  | 0.0845 | 0.0542 |  | 0.0306 | 0.0185 |  | 0.0101 | 0.0205 |  | -0.0161 | 0.0203 |
| 91 | 3 | rs1797625 | 114,309,105 | C3orf17 | T | A | 0.0190 | 0.0030 |  | 0.0674 | 0.0536 |  | -0.0038 | 0.0182 |  | 0.0127 | 0.0191 |  | 0.0034 | 0.0197 |
| 92 | 3 | rs1546391 | 116,180,147 | ZBTB20 | G | C | 0.0392 | 0.0060 |  | -0.1529 | 0.0867 |  | -0.0102 | 0.0355 |  | -0.0515 | 0.0367 |  | 0.0572 | 0.0326 |
| 93 | 3 | rs6439168 | 130,533,633 | H1FX | G | A | 0.0367 | 0.0040 |  | 0.0243 | 0.0627 |  | -0.0150 | 0.0214 |  | -0.0484 | 0.0263 |  | -0.0234 | 0.0210 |
| 94 | 3 | rs4974480 | 135,661,252 | ANAPC13 | T | A | 0.0263 | 0.0030 |  | 0.0742 | 0.0539 |  | -0.0145 | 0.0186 |  | -0.0001 | 0.0199 |  | -0.0055 | 0.0200 |
| 95 | 3 | rs9880211 | 137,590,239 | STAG1 | G | A | 0.0298 | 0.0030 |  | . | . |  | 0.0510 | 0.0201 |  | -0.0082 | 0.0216 |  | 0.0349 | 0.0197 |
| 96 | 3 | rs724016 | 142,588,260 | ZBTB38 | G | A | 0.0779 | 0.0030 |  | -0.0536 | 0.0513 |  | 0.0455 | 0.0175 |  | 0.0891 | 0.0181 |  | -0.0097 | 0.0173 |
| 97 | 3 | rs936339 | 144,018,195 | PCOLCE2 | T | C | 0.0217 | 0.0040 |  | -0.0512 | 0.0680 |  | 0.0185 | 0.0221 |  | 0.0141 | 0.0260 |  | 0.0186 | 0.0221 |
| 98 | 3 | rs6441170 | 159,289,654 | SHOX2 | C | T | 0.0215 | 0.0030 |  | 0.0780 | 0.0528 |  | 0.0051 | 0.0178 |  | -0.0107 | 0.0193 |  | 0.0056 | 0.0175 |
| 99 | 3 | rs7652177 | 173,451,771 | FNDC3B | G | C | 0.0384 | 0.0030 |  | . | . |  | 0.0003 | 0.0179 |  | -0.0106 | 0.0213 |  | 0.0058 | 0.0266 |
| 100 | 3 | rs9858528 | 184,838,099 | KLHL24 | A | G | 0.0221 | 0.0030 |  | 0.0294 | 0.0572 |  | 0.0024 | 0.0198 |  | -0.0349 | 0.0219 |  | -0.0002 | 0.0196 |
| 101 | 3 | rs720390 | 187,031,377 | IGF2BP2 | A | G | 0.0351 | 0.0030 |  | 0.0493 | 0.0534 |  | -0.0463 | 0.0193 |  | -0.0110 | 0.0192 |  | -0.0083 | 0.0180 |
| 102 | 3 | rs4686904 | 188,921,216 | BCL6 | C | T | 0.0209 | 0.0030 |  | -0.0145 | 0.0535 |  | -0.0058 | 0.0188 |  | 0.0223 | 0.0202 |  | -0.0151 | 0.0180 |
| 103 | 3 | rs9841435 | 192,593,854 | CCDC50 | G | A | 0.0197 | 0.0030 |  | 0.0853 | 0.0538 |  | -0.0127 | 0.0185 |  | 0.0045 | 0.0211 |  | 0.0254 | 0.0181 |
| 104 | 4 | rs3958122 | 1,663,729 | SLBP | T | C | 0.0271 | 0.0030 |  | -0.0110 | 0.0541 |  | -0.0081 | 0.0185 |  | -0.0196 | 0.0227 |  | -0.0059 | 0.0196 |
| 105 | 4 | rs6446315 | 5,086,488 | CYTL1 | G | A | 0.0283 | 0.0040 |  | . | . |  | -0.0140 | 0.0275 |  | 0.0035 | 0.0269 |  | -0.0075 | 0.0232 |
| 106 | 4 | rs2302580 | 8,659,534 | CPZ | C | T | 0.0285 | 0.0040 |  | 0.0200 | 0.0521 |  | 0.0080 | 0.0205 |  | 0.0298 | 0.0207 |  | 0.0271 | 0.0192 |
| 107 | 4 | rs763318 | 12,572,672 | RAB28 | G | A | 0.0210 | 0.0030 |  | 0.0004 | 0.0510 |  | 0.0145 | 0.0172 |  | 0.0201 | 0.0197 |  | -0.0035 | 0.0171 |
| 108 | 4 | rs7692995 | 17,545,732 | LCORL | T | C | 0.0743 | 0.0040 |  | -0.0489 | 0.0663 |  | -0.0324 | 0.0236 |  | 0.0063 | 0.0280 |  | -0.0378 | 0.0238 |
| 109 | 4 | rs2306596 | 39,020,335 | RFC1 | A | C | 0.0194 | 0.0030 |  | -0.0272 | 0.0512 |  | -0.0059 | 0.0175 |  | 0.0242 | 0.0215 |  | 0.0208 | 0.0170 |
| 110 | 4 | rs1996422 | 48,382,108 | FRYL | G | A | 0.0220 | 0.0030 |  | 0.0233 | 0.0583 |  | 0.0431 | 0.0196 |  | -0.0228 | 0.0208 |  | 0.0077 | 0.0191 |
| 111 | 4 | rs13113518 | 56,094,405 | CLOCK | C | T | 0.0175 | 0.0030 |  | 0.0745 | 0.0528 |  | -0.0294 | 0.0178 |  | 0.0248 | 0.0221 |  | 0.0151 | 0.0176 |
| 112 | 4 | rs17081935 | 57,518,233 | C4orf14 | T | C | 0.0308 | 0.0040 |  | 0.0676 | 0.0657 |  | 0.0203 | 0.0219 |  | -0.0063 | 0.0239 |  | 0.0100 | 0.0213 |
| 113 | 4 | rs9993613 | 73,694,878 | ADAMTS3 | T | G | 0.0298 | 0.0030 |  | -0.0307 | 0.0510 |  | 0.0054 | 0.0178 |  | 0.0260 | 0.0188 |  | 0.0062 | 0.0174 |
| 114 | 4 | rs17556750 | 82,374,592 | PRKG2 | A | C | 0.0462 | 0.0030 |  | . | . |  | -0.0233 | 0.0189 |  | 0.0346 | 0.0235 |  | 0.0071 | 0.0191 |
| 115 | 4 | rs6813055 | 88,849,055 | DMP1 | A | T | 0.0165 | 0.0030 |  | -0.0089 | 0.0530 |  | 0.0191 | 0.0175 |  | 0.0001 | 0.0197 |  | 0.0091 | 0.0265 |
| 116 | 4 | rs12639764 | 106,435,654 | TET2 | T | C | 0.0274 | 0.0030 |  | -0.0264 | 0.0524 |  | 0.0523 | 0.0179 |  | -0.0393 | 0.0186 |  | 0.0225 | 0.0176 |
| 117 | 4 | rs1562975 | 109,628,057 | RPL34 | A | G | 0.0251 | 0.0030 |  | -0.0500 | 0.0550 |  | -0.0101 | 0.0189 |  | 0.0191 | 0.0214 |  | -0.0315 | 0.0190 |
| 118 | 4 | rs7659107 | 114,961,698 | CAMK2D | G | A | 0.0241 | 0.0040 |  | -0.0030 | 0.0605 |  | 0.0126 | 0.0210 |  | 0.0097 | 0.0255 |  | 0.0038 | 0.0210 |
| 119 | 4 | rs6838153 | 122,940,449 | EXOSC9 | G | A | 0.0215 | 0.0030 |  | 0.0087 | 0.0542 |  | -0.0078 | 0.0182 |  | 0.0156 | 0.0206 |  | -0.0188 | 0.0179 |
| 120 | 4 | rs12513181 | 124,055,106 | NUDT6 | C | A | 0.0196 | 0.0030 |  | 0.1062 | 0.0583 |  | 0.0074 | 0.0196 |  | 0.0053 | 0.0223 |  | -0.0074 | 0.0194 |
| 121 | 4 | rs996743 | 146,348,334 | OTUD4 | A | G | 0.0633 | 0.0070 |  | . | . |  | 0.0752 | 0.0390 |  | -0.0030 | 0.0381 |  | -0.0176 | 0.0331 |
| 122 | 4 | rs13150868 | 152,400,121 | ESSPL | T | G | 0.0169 | 0.0030 |  | -0.0757 | 0.0511 |  | -0.0094 | 0.0175 |  | -0.0121 | 0.0192 |  | 0.0100 | 0.0192 |
| 123 | 4 | rs955748 | 184,452,669 | WWC2 | G | A | 0.0276 | 0.0030 |  | 0.0255 | 0.0596 |  | 0.0156 | 0.0201 |  | -0.0048 | 0.0230 |  | 0.0247 | 0.0199 |
| 124 | 5 | rs17410035 | 31,576,899 | C5orf22 | T | G | 0.0192 | 0.0030 |  | -0.0083 | 0.0553 |  | 0.0066 | 0.0186 |  | -0.0556 | 0.0214 |  | -0.0049 | 0.0181 |
| 125 | 5 | rs9292468 | 32,854,830 | C5orf23 | T | C | 0.0359 | 0.0030 |  | 0.0715 | 0.0509 |  | -0.0070 | 0.0175 |  | -0.0268 | 0.0218 |  | -0.0059 | 0.0173 |
| 126 | 5 | rs301901 | 37,082,383 | NIPBL | A | G | 0.0239 | 0.0030 |  | -0.0507 | 0.0516 |  | 0.0027 | 0.0174 |  | -0.0169 | 0.0190 |  | 0.0284 | 0.0170 |
| 127 | 5 | rs3812040 | 39,461,777 | DAB2 | T | C | 0.0240 | 0.0030 |  | 0.0434 | 0.0568 |  | -0.0153 | 0.0207 |  | 0.0275 | 0.0240 |  | -0.0173 | 0.0194 |
| 128 | 5 | rs17574650 | 42,472,673 | GHR | C | A | 0.0382 | 0.0050 |  | . | . |  | -0.0014 | 0.0320 |  | -0.0637 | 0.0328 |  | 0.0152 | 0.0343 |
| 129 | 5 | rs2961830 | 50,490,489 | ISL1 | A | T | 0.0197 | 0.0030 |  | -0.0020 | 0.0539 |  | -0.0124 | 0.0180 |  | -0.0029 | 0.0194 |  | 0.0138 | 0.0178 |
| 130 | 5 | rs7716219 | 54,990,828 | SLC38A9 | T | C | 0.0302 | 0.0030 |  | -0.2138 | 0.0555 |  | -0.0065 | 0.0188 |  | 0.0277 | 0.0208 |  | 0.0243 | 0.0187 |
| 131 | 5 | rs2662027 | 56,290,242 | MIER3 | G | T | 0.0332 | 0.0050 |  | -0.0927 | 0.0849 |  | -0.0032 | 0.0288 |  | 0.0031 | 0.0324 |  | -0.0261 | 0.0278 |
| 132 | 5 | rs7727731 | 64,710,202 | ADAMTS6 | T | C | 0.0334 | 0.0050 |  | . | . |  | 0.0027 | 0.0295 |  | 0.0160 | 0.0291 |  | -0.0282 | 0.0283 |
| 133 | 5 | rs9291926 | 67,635,412 | PIK3R1 | T | G | 0.0192 | 0.0030 |  | 0.0945 | 0.0510 |  | 0.0157 | 0.0178 |  | 0.0008 | 0.0193 |  | 0.0295 | 0.0171 |
| 134 | 5 | rs34651 | 72,179,761 | TNPO1 | C | T | 0.0407 | 0.0060 |  | -0.0257 | 0.0970 |  | -0.0127 | 0.0348 |  | 0.0513 | 0.0359 |  | 0.0470 | 0.0381 |
| 135 | 5 | rs820848 | 74,000,416 | HEXB | G | A | 0.0206 | 0.0040 |  | 0.0155 | 0.0572 |  | -0.0440 | 0.0218 |  | -0.0013 | 0.0232 |  | 0.0028 | 0.0217 |
| 136 | 5 | rs12519505 | 77,541,632 | AP3B1 | C | T | 0.0221 | 0.0040 |  | 0.0827 | 0.0608 |  | -0.0138 | 0.0207 |  | 0.0241 | 0.0256 |  | -0.0169 | 0.0209 |
| 137 | 5 | rs32855 | 79,871,948 | FAM151B | A | G | 0.0235 | 0.0040 |  | 0.0178 | 0.0628 |  | 0.0065 | 0.0212 |  | -0.0011 | 0.0241 |  | 0.0166 | 0.0208 |
| 138 | 5 | rs6894139 | 88,363,538 | MEF2C | T | G | 0.0304 | 0.0030 |  | 0.0078 | 0.0509 |  | 0.0360 | 0.0177 |  | -0.0435 | 0.0213 |  | 0.0181 | 0.0171 |
| 139 | 5 | rs2247870 | 90,187,345 | GPR98 | A | G | 0.0159 | 0.0030 |  | . | . |  | 0.0162 | 0.0175 |  | 0.0118 | 0.0189 |  | -0.0251 | 0.0170 |
| 140 | 5 | rs12186664 | 95,655,981 | PCSK1 | T | A | 0.0210 | 0.0030 |  | 0.0413 | 0.0545 |  | 0.0176 | 0.0185 |  | 0.0256 | 0.0209 |  | 0.0105 | 0.0181 |
| 141 | 5 | rs13177718 | 108,141,243 | FER | C | T | 0.0431 | 0.0060 |  | -0.0939 | 0.0972 |  | 0.0504 | 0.0343 |  | 0.0198 | 0.0376 |  | -0.0122 | 0.0335 |
| 142 | 5 | rs1582931 | 122,685,098 | CCDC100 | G | A | 0.0279 | 0.0030 |  | 0.0130 | 0.0516 |  | 0.0454 | 0.0181 |  | -0.0191 | 0.0198 |  | -0.0177 | 0.0174 |
| 143 | 5 | rs26024 | 127,723,921 | FBN2 | C | A | 0.0234 | 0.0030 |  | -0.0688 | 0.0531 |  | 0.0153 | 0.0180 |  | -0.0348 | 0.0192 |  | -0.0076 | 0.0180 |
| 144 | 5 | rs39623 | 129,082,520 | ADAMTS19 | A | T | 0.0342 | 0.0050 |  | -0.1686 | 0.0995 |  | 0.0041 | 0.0327 |  | 0.0204 | 0.0377 |  | 0.0226 | 0.0308 |
| 145 | 5 | rs7701414 | 131,613,857 | PDLIM4 | G | A | 0.0365 | 0.0030 |  | . | . |  | 0.0368 | 0.0174 |  | 0.0170 | 0.0185 |  | 0.0454 | 0.0173 |
| 146 | 5 | rs526896 | 134,384,604 | PITX1 | T | G | 0.0355 | 0.0030 |  | 0.0047 | 0.0675 |  | -0.0003 | 0.0203 |  | 0.0119 | 0.0249 |  | 0.0219 | 0.0202 |
| 147 | 5 | rs165189 | 139,125,931 | PSD2 | G | A | 0.0294 | 0.0050 |  | 0.0543 | 0.0755 |  | 0.0424 | 0.0268 |  | -0.0104 | 0.0283 |  | 0.0137 | 0.0280 |
| 148 | 5 | rs4624820 | 141,661,972 | SPRY4 | A | G | 0.0179 | 0.0030 |  | 0.0324 | 0.0517 |  | -0.0240 | 0.0172 |  | 0.0099 | 0.0184 |  | -0.0181 | 0.0170 |
| 149 | 5 | rs2974438 | 168,183,481 | SLIT3 | G | A | 0.0369 | 0.0040 |  | -0.0030 | 0.0622 |  | -0.0053 | 0.0213 |  | -0.0208 | 0.0232 |  | 0.0191 | 0.0212 |
| 150 | 5 | rs4868126 | 171,216,074 | FBXW11 | G | T | 0.0356 | 0.0030 |  | . | . |  | -0.0109 | 0.0183 |  | -0.0468 | 0.0221 |  | 0.0102 | 0.0193 |
| 151 | 5 | rs7733195 | 172,927,230 | FAM44B | G | A | 0.0288 | 0.0030 |  | 0.0980 | 0.0525 |  | -0.0124 | 0.0182 |  | -0.0509 | 0.0210 |  | 0.0213 | 0.0178 |
| 152 | 5 | rs422421 | 176,449,932 | FGFR4 | C | T | 0.0338 | 0.0040 |  | 0.0559 | 0.0623 |  | -0.0010 | 0.0212 |  | -0.0105 | 0.0262 |  | -0.0071 | 0.0208 |
| 153 | 5 | rs11750568 | 178,468,319 | ADAMTS2 | A | G | 0.0201 | 0.0030 |  | 0.0769 | 0.0552 |  | -0.0096 | 0.0182 |  | 0.0122 | 0.0225 |  | -0.0070 | 0.0180 |
| 154 | 5 | rs6879260 | 179,663,620 | GFPT2 | C | T | 0.0269 | 0.0030 |  | 0.0910 | 0.0525 |  | 0.0199 | 0.0179 |  | 0.0155 | 0.0198 |  | 0.0152 | 0.0176 |
| 155 | 6 | rs932445 | 2,112,224 | GMDS | T | C | 0.0176 | 0.0030 |  | . | . |  | 0.0205 | 0.0186 |  | -0.0277 | 0.0199 |  | 0.0078 | 0.0183 |
| 156 | 6 | rs9392918 | 7,653,630 | BMP6 | C | T | 0.0381 | 0.0030 |  | 0.0342 | 0.0505 |  | -0.0100 | 0.0176 |  | 0.0227 | 0.0211 |  | 0.0240 | 0.0171 |
| 157 | 6 | rs17330192 | 17,697,354 | FAM8A1 | C | T | 0.0188 | 0.0030 |  | -0.0293 | 0.0606 |  | 0.0264 | 0.0200 |  | -0.0344 | 0.0221 |  | 0.0003 | 0.0196 |
| 158 | 6 | rs1047014 | 19,949,472 | ID4 | C | T | 0.0317 | 0.0040 |  | . | . |  | -0.0071 | 0.0207 |  | 0.0426 | 0.0209 |  | -0.0064 | 0.0197 |
| 159 | 6 | rs4141885 | 26,265,460 | HIST1H1E | A | T | 0.0728 | 0.0080 |  | 0.0395 | 0.0832 |  | -0.0172 | 0.0407 |  | 0.0547 | 0.0325 |  | 0.0679 | 0.0319 |
| 160 | 6 | rs1233627 | 28,859,706 | TRIM27 | T | C | 0.0238 | 0.0030 |  | 0.0295 | 0.0514 |  | -0.0127 | 0.0174 |  | . | . |  | -0.0441 | 0.0221 |
| 161 | 6 | rs9404952 | 29,912,144 | HLA-G | A | G | 0.0177 | 0.0030 |  | 0.0056 | 0.0515 |  | 0.0222 | 0.0175 |  | . | . |  | -0.0221 | 0.0225 |
| 162 | 6 | rs6457374 | 31,380,240 | HLA-C | C | T | 0.0414 | 0.0030 |  | -0.0383 | 0.0682 |  | 0.0221 | 0.0204 |  | . | . |  | 0.0867 | 0.0294 |
| 163 | 6 | rs12214804 | 34,296,844 | HMGA1 | C | T | 0.0842 | 0.0060 |  | 0.0871 | 0.0850 |  | -0.0156 | 0.0323 |  | 0.0152 | 0.0389 |  | -0.0064 | 0.0321 |
| 164 | 6 | rs16895130 | 42,032,909 | CCND3 | G | A | 0.0227 | 0.0030 |  | 0.0743 | 0.0559 |  | -0.0286 | 0.0195 |  | 0.0074 | 0.0209 |  | 0.0142 | 0.0190 |
| 165 | 6 | rs10948222 | 45,352,393 | SUPT3H | C | T | 0.0311 | 0.0030 |  | 0.0621 | 0.0520 |  | 0.0154 | 0.0185 |  | 0.0091 | 0.0201 |  | 0.0183 | 0.0179 |
| 166 | 6 | rs9395264 | 47,582,981 | CD2AP | G | T | 0.0199 | 0.0030 |  | -0.0421 | 0.0548 |  | -0.0046 | 0.0187 |  | -0.0366 | 0.0215 |  | -0.0107 | 0.0183 |
| 167 | 6 | rs12190423 | 72,259,432 | OGFRL1 | G | C | 0.0166 | 0.0030 |  | -0.0410 | 0.0534 |  | 0.0189 | 0.0177 |  | 0.0402 | 0.0196 |  | -0.0017 | 0.0174 |
| 168 | 6 | rs12209223 | 76,221,309 | FILIP1 | A | C | 0.0505 | 0.0050 |  | . | . |  | 0.0034 | 0.0291 |  | -0.0163 | 0.0343 |  | -0.0418 | 0.0294 |
| 169 | 6 | rs310421 | 81,848,782 | FAM46A | T | G | 0.0318 | 0.0030 |  | . | . |  | -0.0272 | 0.0174 |  | 0.0025 | 0.0192 |  | -0.0087 | 0.0180 |
| 170 | 6 | rs761391 | 85,504,822 | TBX18 | C | T | 0.0193 | 0.0030 |  | 0.0178 | 0.0515 |  | -0.0222 | 0.0187 |  | -0.0414 | 0.0190 |  | 0.0278 | 0.0180 |
| 171 | 6 | rs314263 | 105,499,438 | LIN28B | C | T | 0.0427 | 0.0030 |  | . | . |  | -0.0154 | 0.0188 |  | -0.0015 | 0.0192 |  | -0.0178 | 0.0182 |
| 172 | 6 | rs6920372 | 109,830,632 | PPIL6 | G | A | 0.0251 | 0.0030 |  | -0.0555 | 0.0515 |  | -0.0181 | 0.0174 |  | -0.0040 | 0.0216 |  | -0.0267 | 0.0172 |
| 173 | 6 | rs2145357 | 116,558,135 | NT5DC1 | G | A | 0.0209 | 0.0030 |  | 0.0665 | 0.0560 |  | -0.0196 | 0.0196 |  | -0.0323 | 0.0206 |  | 0.0173 | 0.0193 |
| 174 | 6 | rs1405212 | 117,597,357 | VGLL2 | C | T | 0.0231 | 0.0030 |  | . | . |  | 0.0033 | 0.0175 |  | 0.0393 | 0.0192 |  | 0.0190 | 0.0177 |
| 175 | 6 | rs1155939 | 126,907,826 | C6orf173 | A | C | 0.0418 | 0.0030 |  | -0.0554 | 0.0508 |  | 0.0006 | 0.0172 |  | 0.0135 | 0.0197 |  | -0.0133 | 0.0170 |
| 176 | 6 | rs7740107 | 130,416,154 | L3MBTL3 | T | A | 0.0421 | 0.0030 |  | 0.0275 | 0.0596 |  | -0.0780 | 0.0198 |  | -0.0787 | 0.0228 |  | 0.0158 | 0.0193 |
| 177 | 6 | rs7743622 | 132,772,065 | MOXD1 | G | C | 0.0172 | 0.0030 |  | 0.0375 | 0.0517 |  | -0.0337 | 0.0194 |  | -0.0461 | 0.0209 |  | 0.0165 | 0.0239 |
| 178 | 6 | rs4896582 | 142,745,570 | GPR126 | G | A | 0.0509 | 0.0030 |  | 0.0418 | 0.0550 |  | -0.0074 | 0.0190 |  | 0.0395 | 0.0234 |  | 0.0023 | 0.0187 |
| 179 | 6 | rs6911389 | 144,121,322 | PHACTR2 | T | G | 0.0147 | 0.0030 |  | -0.0780 | 0.0546 |  | -0.0429 | 0.0184 |  | 0.0388 | 0.0226 |  | 0.0055 | 0.0180 |
| 180 | 6 | rs2748483 | 146,377,253 | GRM1 | A | T | 0.0185 | 0.0030 |  | 0.0381 | 0.0522 |  | -0.0310 | 0.0173 |  | -0.0061 | 0.0214 |  | -0.0169 | 0.0268 |
| 181 | 6 | rs6902771 | 152,199,574 | ESR1 | T | C | 0.0311 | 0.0030 |  | 0.0118 | 0.0511 |  | -0.0248 | 0.0178 |  | -0.0196 | 0.0217 |  | -0.0100 | 0.0173 |
| 182 | 6 | rs11156098 | 156,629,523 | ARID1B | T | C | 0.0266 | 0.0050 |  | -0.0854 | 0.0894 |  | -0.0391 | 0.0289 |  | 0.0158 | 0.0323 |  | 0.0245 | 0.0262 |
| 183 | 6 | rs1832871 | 158,642,022 | TULP4 | A | G | 0.0245 | 0.0030 |  | 0.0381 | 0.0547 |  | 0.0174 | 0.0181 |  | -0.0076 | 0.0210 |  | -0.0241 | 0.0179 |
| 184 | 6 | rs486359 | 160,694,431 | SLC22A3 | C | G | 0.0158 | 0.0030 |  | 0.0206 | 0.0515 |  | 0.0131 | 0.0176 |  | -0.1023 | 0.0180 |  | 0.0502 | 0.0354 |
| 185 | 6 | rs991946 | 166,249,852 | T | C | T | 0.0209 | 0.0030 |  | 0.0880 | 0.0520 |  | 0.0258 | 0.0172 |  | -0.0065 | 0.0202 |  | 0.0198 | 0.0171 |
| 186 | 6 | rs2763273 | 168,577,472 | SMOC2 | C | T | 0.0216 | 0.0030 |  | -0.0144 | 0.0615 |  | -0.0145 | 0.0205 |  | -0.0383 | 0.0234 |  | 0.0023 | 0.0198 |
| 187 | 7 | rs798497 | 2,762,483 | GNA12 | A | G | 0.0569 | 0.0030 |  | . | . |  | -0.0032 | 0.0186 |  | -0.0283 | 0.0218 |  | 0.0293 | 0.0186 |
| 188 | 7 | rs4725061 | 8,053,164 | GLCCI1 | G | A | 0.0199 | 0.0030 |  | . | . |  | -0.0289 | 0.0183 |  | 0.0249 | 0.0205 |  | -0.0052 | 0.0200 |
| 189 | 7 | rs929637 | 12,243,047 | TMEM106B | G | T | 0.0212 | 0.0040 |  | . | . |  | 0.0048 | 0.0206 |  | -0.0163 | 0.0232 |  | -0.0252 | 0.0204 |
| 190 | 7 | rs3807931 | 20,348,199 | ITGB8 | A | G | 0.0267 | 0.0030 |  | . | . |  | 0.0002 | 0.0173 |  | -0.0324 | 0.0198 |  | -0.0231 | 0.0170 |
| 191 | 7 | rs12538407 | 23,487,841 | IGF2BP3 | A | G | 0.0322 | 0.0030 |  | . | . |  | 0.0028 | 0.0182 |  | -0.0277 | 0.0193 |  | -0.0164 | 0.0177 |
| 192 | 7 | rs1055144 | 25,837,634 | NFE2L3 | T | C | 0.0214 | 0.0040 |  | . | . |  | -0.0332 | 0.0220 |  | -0.0662 | 0.0248 |  | 0.0026 | 0.0216 |
| 193 | 7 | rs552707 | 28,171,828 | JAZF1 | T | C | 0.0458 | 0.0030 |  | . | . |  | -0.0075 | 0.0194 |  | 0.0124 | 0.0199 |  | -0.0302 | 0.0189 |
| 194 | 7 | rs6462432 | 32,902,049 | KBTBD2 | A | G | 0.0166 | 0.0030 |  | . | . |  | -0.0203 | 0.0176 |  | 0.0015 | 0.0199 |  | 0.0154 | 0.0176 |
| 195 | 7 | rs6974574 | 38,076,598 | STARD3NL | T | A | 0.0303 | 0.0030 |  | . | . |  | 0.0072 | 0.0202 |  | 0.0337 | 0.0228 |  | 0.0133 | 0.0195 |
| 196 | 7 | rs6949739 | 46,383,928 | IGFBP3 | T | A | 0.0380 | 0.0050 |  | . | . |  | 0.0108 | 0.0316 |  | -0.0306 | 0.0364 |  | 0.0524 | 0.0314 |
| 197 | 7 | rs2715094 | 50,697,946 | GRB10 | G | A | 0.0209 | 0.0030 |  | . | . |  | 0.0118 | 0.0202 |  | -0.0016 | 0.0246 |  | -0.0018 | 0.0199 |
| 198 | 7 | rs1113765 | 55,856,828 | 14-Sep | G | A | 0.0241 | 0.0040 |  | . | . |  | 0.0318 | 0.0219 |  | -0.0112 | 0.0277 |  | -0.0583 | 0.0242 |
| 199 | 7 | rs12669267 | 72,942,572 | WBSCR28 | C | T | 0.0286 | 0.0050 |  | . | . |  | -0.0331 | 0.0280 |  | -0.0179 | 0.0318 |  | -0.0239 | 0.0265 |
| 200 | 7 | rs17807185 | 77,146,231 | RSBN1L | G | A | 0.0217 | 0.0030 |  | . | . |  | 0.0234 | 0.0176 |  | 0.0116 | 0.0207 |  | -0.0208 | 0.0175 |
| 201 | 7 | rs42039 | 92,082,358 | CDK6 | T | C | 0.0683 | 0.0030 |  | . | . |  | 0.0115 | 0.0200 |  | 0.0172 | 0.0230 |  | -0.0008 | 0.0203 |
| 202 | 7 | rs6971575 | 95,877,584 | SLC25A13 | C | G | 0.0210 | 0.0040 |  | . | . |  | -0.0013 | 0.0212 |  | 0.0143 | 0.0219 |  | -0.0140 | 0.0210 |
| 203 | 7 | rs17250196 | 99,655,132 | GATS/PVRIG | T | G | 0.0449 | 0.0070 |  | . | . |  | 0.0624 | 0.0403 |  | 0.0669 | 0.0426 |  | 0.0226 | 0.0433 |
| 204 | 7 | rs6952113 | 120,564,855 | C7orf58 | G | A | 0.0183 | 0.0030 |  | . | . |  | -0.0083 | 0.0179 |  | 0.0327 | 0.0184 |  | -0.0176 | 0.0174 |
| 205 | 7 | rs6962887 | 134,696,326 | CNOT4 | T | G | 0.0225 | 0.0030 |  | . | . |  | 0.0304 | 0.0218 |  | 0.0313 | 0.0236 |  | -0.0134 | 0.0201 |
| 206 | 7 | rs273945 | 137,262,106 | CREB3L2 | C | A | 0.0189 | 0.0030 |  | . | . |  | -0.0025 | 0.0181 |  | -0.0067 | 0.0198 |  | -0.0010 | 0.0177 |
| 207 | 7 | rs822531 | 148,260,692 | EZH2 | T | C | 0.0355 | 0.0040 |  | . | . |  | -0.0282 | 0.0234 |  | 0.0449 | 0.0271 |  | 0.0203 | 0.0237 |
| 208 | 7 | rs6955948 | 150,139,653 | TMEM176A | T | C | 0.0311 | 0.0030 |  | . | . |  | -0.0420 | 0.0199 |  | 0.0305 | 0.0201 |  | -0.0232 | 0.0212 |
| 209 | 8 | rs4875421 | 4,814,740 | CSMD1 | T | A | 0.0188 | 0.0030 |  | -0.0670 | 0.0504 |  | -0.0002 | 0.0174 |  | 0.0085 | 0.0214 |  | -0.0410 | 0.0268 |
| 210 | 8 | rs429433 | 8,785,304 | MFHAS1 | A | G | 0.0459 | 0.0070 |  | -0.0514 | 0.1362 |  | 0.0434 | 0.0416 |  | 0.0384 | 0.0434 |  | -0.0343 | 0.0416 |
| 211 | 8 | rs7834383 | 13,317,848 | DLC1 | T | G | 0.0215 | 0.0030 |  | 0.0088 | 0.0559 |  | 0.0294 | 0.0187 |  | 0.0183 | 0.0196 |  | 0.0018 | 0.0186 |
| 212 | 8 | rs2013265 | 24,148,445 | ADAM28 | C | T | 0.0275 | 0.0030 |  | . | . |  | 0.0343 | 0.0199 |  | -0.0394 | 0.0232 |  | 0.0166 | 0.0193 |
| 213 | 8 | rs3812423 | 25,354,627 | KCTD9 | G | C | 0.0214 | 0.0030 |  | 0.0275 | 0.0530 |  | -0.0120 | 0.0179 |  | 0.0116 | 0.0221 |  | 0.0168 | 0.0175 |
| 214 | 8 | rs568610 | 27,583,914 | SCARA3 | T | C | 0.0220 | 0.0030 |  | . | . |  | 0.0058 | 0.0200 |  | -0.0059 | 0.0212 |  | 0.0049 | 0.0202 |
| 215 | 8 | rs6988484 | 49,576,333 | EFCAB1 | C | T | 0.0220 | 0.0030 |  | -0.0163 | 0.0563 |  | -0.0130 | 0.0196 |  | -0.0093 | 0.0208 |  | 0.0228 | 0.0193 |
| 216 | 8 | rs9650315 | 57,318,152 | CHCHD7 | G | T | 0.0611 | 0.0050 |  | 0.0730 | 0.0732 |  | -0.0295 | 0.0261 |  | -0.0216 | 0.0307 |  | -0.0484 | 0.0256 |
| 217 | 8 | rs2956605 | 76,045,609 | CRISPLD1 | A | C | 0.0242 | 0.0030 |  | 0.0093 | 0.0520 |  | -0.0071 | 0.0183 |  | -0.0055 | 0.0206 |  | -0.0152 | 0.0179 |
| 218 | 8 | rs4735677 | 78,310,746 | PXMP3 | T | A | 0.0369 | 0.0030 |  | -0.0206 | 0.0570 |  | 0.0029 | 0.0190 |  | 0.0192 | 0.0213 |  | 0.0324 | 0.0189 |
| 219 | 8 | rs7007200 | 109,854,114 | TMEM74 | G | C | 0.0170 | 0.0030 |  | 0.0251 | 0.0561 |  | -0.0111 | 0.0190 |  | -0.0078 | 0.0217 |  | 0.0355 | 0.0184 |
| 220 | 8 | rs1550162 | 117,632,713 | EIF3H | G | A | 0.0240 | 0.0030 |  | . | . |  | 0.0241 | 0.0194 |  | -0.0211 | 0.0236 |  | 0.0433 | 0.0188 |
| 221 | 8 | rs1599473 | 120,544,539 | NOV | G | T | 0.0266 | 0.0030 |  | -0.0280 | 0.0595 |  | -0.0262 | 0.0205 |  | 0.0061 | 0.0253 |  | 0.0158 | 0.0213 |
| 222 | 8 | rs11779459 | 124,049,732 | ZHX2 | T | C | 0.0163 | 0.0030 |  | 0.0778 | 0.0531 |  | 0.0069 | 0.0190 |  | -0.0140 | 0.0227 |  | -0.0009 | 0.0196 |
| 223 | 8 | rs8180991 | 126,569,532 | TRIB1 | C | G | 0.0284 | 0.0040 |  | 0.0586 | 0.0622 |  | -0.0282 | 0.0211 |  | -0.0190 | 0.0255 |  | 0.0234 | 0.0206 |
| 224 | 8 | rs4733724 | 130,792,910 | MLZE | A | G | 0.0504 | 0.0040 |  | 0.0716 | 0.0636 |  | 0.0137 | 0.0218 |  | 0.0123 | 0.0230 |  | 0.0123 | 0.0216 |
| 225 | 8 | rs1036821 | 135,719,665 | ZFAT | G | A | 0.0371 | 0.0030 |  | . | . |  | 0.0163 | 0.0190 |  | -0.0098 | 0.0213 |  | 0.0121 | 0.0185 |
| 226 | 8 | rs11783655 | 145,109,561 | PLEC1 | T | A | 0.0183 | 0.0030 |  | 0.0825 | 0.0516 |  | -0.0184 | 0.0177 |  | -0.0171 | 0.0219 |  | -0.0474 | 0.0227 |
| 227 | 9 | rs7033940 | 6,430,419 | UHRF2 | G | C | 0.0242 | 0.0040 |  | 0.1126 | 0.0768 |  | 0.0156 | 0.0257 |  | -0.0474 | 0.0283 |  | 0.0167 | 0.0249 |
| 228 | 9 | rs2149163 | 16,445,833 | BNC2 | C | G | 0.0202 | 0.0030 |  | -0.0095 | 0.0529 |  | -0.0205 | 0.0178 |  | -0.0088 | 0.0192 |  | 0.0172 | 0.0195 |
| 229 | 9 | rs1576900 | 18,619,792 | ADAMTSL1 | G | A | 0.0190 | 0.0030 |  | -0.0013 | 0.0558 |  | -0.0070 | 0.0191 |  | 0.0163 | 0.0202 |  | -0.0044 | 0.0187 |
| 230 | 9 | rs3763631 | 35,798,334 | NPR2/SPAG8 | C | G | 0.0193 | 0.0030 |  | -0.0113 | 0.0571 |  | -0.0173 | 0.0186 |  | 0.0259 | 0.0216 |  | 0.0326 | 0.0183 |
| 231 | 9 | rs11144688 | 77,732,106 | PCSK5 | G | A | 0.0633 | 0.0060 |  | 0.1128 | 0.0785 |  | 0.0896 | 0.0567 |  | -0.0201 | 0.0472 |  | -0.0067 | 0.0342 |
| 232 | 9 | rs7853235 | 85,850,602 | RMI1 | T | C | 0.0288 | 0.0040 |  | -0.0882 | 0.0651 |  | 0.0302 | 0.0221 |  | -0.0285 | 0.0245 |  | 0.0073 | 0.0217 |
| 233 | 9 | rs181338 | 88,297,981 | ZCCHC6 | T | C | 0.0284 | 0.0030 |  | 0.0028 | 0.0519 |  | 0.0513 | 0.0173 |  | -0.0117 | 0.0201 |  | -0.0058 | 0.0171 |
| 234 | 9 | rs10780910 | 90,039,075 | SPIN1 | T | A | 0.0267 | 0.0030 |  | -0.0053 | 0.0517 |  | 0.0075 | 0.0178 |  | 0.0124 | 0.0191 |  | 0.0465 | 0.0272 |
| 235 | 9 | rs1571892 | 93,298,657 | NFIL3 | C | A | 0.0159 | 0.0030 |  | 0.0496 | 0.0563 |  | 0.0086 | 0.0193 |  | 0.0161 | 0.0237 |  | 0.0008 | 0.0190 |
| 236 | 9 | rs7043114 | 94,427,804 | IPPK | C | T | 0.0285 | 0.0030 |  | 0.1087 | 0.0517 |  | -0.0290 | 0.0174 |  | 0.0310 | 0.0199 |  | 0.0099 | 0.0171 |
| 237 | 9 | rs817300 | 97,420,043 | PTCH1 | G | A | 0.0846 | 0.0070 |  | 0.1379 | 0.1086 |  | 0.0029 | 0.0389 |  | -0.0513 | 0.0396 |  | 0.0117 | 0.0362 |
| 238 | 9 | rs953199 | 99,522,797 | XPA | C | A | 0.0185 | 0.0040 |  | -0.0173 | 0.0576 |  | -0.0318 | 0.0200 |  | 0.0380 | 0.0228 |  | 0.0090 | 0.0200 |
| 239 | 9 | rs989393 | 100,783,157 | COL15A1 | T | C | 0.0215 | 0.0030 |  | 0.0503 | 0.0551 |  | -0.0149 | 0.0193 |  | 0.0377 | 0.0218 |  | 0.0025 | 0.0189 |
| 240 | 9 | rs7027110 | 108,638,867 | ZNF462 | A | G | 0.0319 | 0.0030 |  | -0.0692 | 0.0621 |  | 0.0094 | 0.0204 |  | 0.0340 | 0.0226 |  | 0.0046 | 0.0204 |
| 241 | 9 | rs3739707 | 112,832,527 | LPAR1 | C | A | 0.0240 | 0.0040 |  | 0.0156 | 0.0585 |  | -0.0070 | 0.0203 |  | -0.0035 | 0.0230 |  | -0.0186 | 0.0197 |
| 242 | 9 | rs999599 | 116,051,416 | COL27A1 | T | C | 0.0162 | 0.0030 |  | 0.0565 | 0.1060 |  | 0.0063 | 0.0182 |  | -0.0055 | 0.0205 |  | -0.0033 | 0.0174 |
| 243 | 9 | rs7033487 | 118,169,078 | PAPPA | T | C | 0.0370 | 0.0040 |  | 0.0926 | 0.0649 |  | 0.0264 | 0.0217 |  | 0.0084 | 0.0250 |  | 0.0166 | 0.0210 |
| 244 | 9 | rs7466269 | 132,453,905 | FUBP3 | A | G | 0.0333 | 0.0030 |  | -0.0394 | 0.0540 |  | 0.0106 | 0.0180 |  | -0.0010 | 0.0208 |  | 0.0072 | 0.0176 |
| 245 | 9 | rs3132297 | 136,441,687 | RXRA | G | A | 0.0233 | 0.0040 |  | -0.0023 | 0.0673 |  | 0.0003 | 0.0235 |  | -0.0109 | 0.0261 |  | -0.0256 | 0.0248 |
| 246 | 9 | rs7849585 | 138,251,691 | QSOX2 | T | G | 0.0360 | 0.0030 |  | -0.0598 | 0.0535 |  | 0.0282 | 0.0188 |  | 0.0170 | 0.0230 |  | 0.0175 | 0.0183 |
| 247 | 10 | rs4332428 | 4,955,434 | AKR1C1 | A | G | 0.0360 | 0.0050 |  | 0.0499 | 0.0778 |  | 0.0117 | 0.0267 |  | 0.0305 | 0.0328 |  | 0.0119 | 0.0257 |
| 248 | 10 | rs12779328 | 12,983,979 | CCDC3 | C | T | 0.0281 | 0.0030 |  | . | . |  | -0.0253 | 0.0194 |  | 0.0049 | 0.0215 |  | 0.0128 | 0.0191 |
| 249 | 10 | rs4350272 | 25,096,124 | ARHGAP21 | A | G | 0.0198 | 0.0030 |  | -0.0361 | 0.0592 |  | 0.0190 | 0.0200 |  | -0.0133 | 0.0215 |  | 0.0217 | 0.0195 |
| 250 | 10 | rs7069985 | 27,930,837 | RAB18 | G | A | 0.0230 | 0.0030 |  | -0.0124 | 0.0610 |  | -0.0292 | 0.0207 |  | 0.0024 | 0.0260 |  | 0.0317 | 0.0204 |
| 251 | 10 | rs10995319 | 52,432,893 | PRKG1 | T | C | 0.0189 | 0.0030 |  | -0.0587 | 0.0588 |  | 0.0296 | 0.0203 |  | 0.0268 | 0.0253 |  | 0.0185 | 0.0201 |
| 252 | 10 | rs1171615 | 61,139,096 | SLC16A9 | C | T | 0.0221 | 0.0040 |  | 0.0042 | 0.0599 |  | -0.0268 | 0.0217 |  | -0.0490 | 0.0263 |  | 0.0263 | 0.0206 |
| 253 | 10 | rs10997979 | 69,607,198 | MYPN | G | A | 0.0213 | 0.0030 |  | . | . |  | -0.0183 | 0.0172 |  | 0.0296 | 0.0198 |  | -0.0150 | 0.0170 |
| 254 | 10 | rs1923367 | 80,802,835 | ZCCHC24 | G | C | 0.0299 | 0.0030 |  | -0.0117 | 0.0514 |  | 0.0171 | 0.0174 |  | 0.0222 | 0.0213 |  | 0.0003 | 0.0270 |
| 255 | 10 | rs2631676 | 93,027,389 | PCGF5 | G | A | 0.0282 | 0.0040 |  | 0.0602 | 0.0701 |  | 0.0085 | 0.0233 |  | -0.0121 | 0.0255 |  | 0.0110 | 0.0220 |
| 256 | 10 | rs915506 | 97,795,064 | CCNJ | G | A | 0.0206 | 0.0030 |  | 0.0716 | 0.0524 |  | 0.0088 | 0.0180 |  | 0.0060 | 0.0222 |  | -0.0226 | 0.0177 |
| 257 | 10 | rs10883563 | 102,674,370 | FAM178A | A | C | 0.0227 | 0.0030 |  | -0.0345 | 0.0522 |  | -0.0260 | 0.0174 |  | -0.0082 | 0.0196 |  | -0.0268 | 0.0171 |
| 258 | 10 | rs7899004 | 104,331,425 | SUFU | T | C | 0.0245 | 0.0030 |  | -0.0498 | 0.0519 |  | 0.0200 | 0.0173 |  | -0.0433 | 0.0193 |  | -0.0115 | 0.0174 |
| 259 | 10 | rs6584575 | 105,567,399 | SH3PXD2A | A | G | 0.0335 | 0.0050 |  | -0.0177 | 0.0803 |  | -0.0019 | 0.0288 |  | 0.0709 | 0.0307 |  | 0.0452 | 0.0281 |
| 260 | 10 | rs291979 | 121,119,787 | GRK5 | A | G | 0.0290 | 0.0040 |  | . | . |  | 0.0325 | 0.0206 |  | -0.0425 | 0.0217 |  | -0.0220 | 0.0208 |
| 261 | 10 | rs1614303 | 123,386,796 | FGFR2 | T | G | 0.0224 | 0.0040 |  | -0.0379 | 0.0666 |  | 0.0088 | 0.0224 |  | -0.0299 | 0.0258 |  | 0.0066 | 0.0221 |
| 262 | 10 | rs10794175 | 126,348,063 | FAM53B | T | G | 0.0204 | 0.0030 |  | -0.1181 | 0.0512 |  | 0.0214 | 0.0174 |  | 0.0080 | 0.0203 |  | 0.0051 | 0.0173 |
| 263 | 11 | rs2272566 | 234,552 | PSMD13 | A | G | 0.0164 | 0.0030 |  | 0.0226 | 0.0501 |  | 0.0175 | 0.0173 |  | 0.0487 | 0.0214 |  | 0.0325 | 0.0171 |
| 264 | 11 | rs2237886 | 2,767,307 | KCNQ1 | T | C | 0.0427 | 0.0050 |  | 0.0578 | 0.0883 |  | -0.0217 | 0.0297 |  | -0.0589 | 0.0356 |  | 0.0176 | 0.0286 |
| 265 | 11 | rs6485978 | 12,634,991 | TEAD1 | C | T | 0.0234 | 0.0030 |  | 0.0329 | 0.0506 |  | 0.0001 | 0.0174 |  | 0.0366 | 0.0188 |  | 0.0200 | 0.0170 |
| 266 | 11 | rs757081 | 17,308,259 | NUCB2 | G | C | 0.0242 | 0.0030 |  | . | . |  | -0.0160 | 0.0187 |  | 0.0030 | 0.0206 |  | 0.0217 | 0.0183 |
| 267 | 11 | rs10767838 | 30,304,503 | C11orf46 | A | G | 0.0253 | 0.0030 |  | 0.0382 | 0.0560 |  | 0.0093 | 0.0192 |  | 0.0049 | 0.0221 |  | -0.0131 | 0.0188 |
| 268 | 11 | rs3802758 | 45,892,611 | PEX16 | A | G | 0.0394 | 0.0070 |  | -0.1193 | 0.0887 |  | 0.0404 | 0.0410 |  | 0.0368 | 0.0351 |  | -0.0404 | 0.0351 |
| 269 | 11 | rs1681630 | 47,925,728 | PTPRJ | T | C | 0.0285 | 0.0030 |  | 0.0279 | 0.0537 |  | 0.0251 | 0.0185 |  | -0.0321 | 0.0227 |  | 0.0063 | 0.0182 |
| 270 | 11 | rs1945237 | 55,986,645 | OR5M9/OR8U8 | C | T | 0.0223 | 0.0050 |  | 0.0270 | 0.1006 |  | -0.0079 | 0.0306 |  | 0.0097 | 0.0365 |  | -0.0060 | 0.0303 |
| 271 | 11 | rs3782089 | 65,093,395 | SSSCA1 | C | T | 0.0534 | 0.0070 |  | 0.0368 | 0.0903 |  | 0.0321 | 0.0370 |  | -0.0385 | 0.0399 |  | 0.0283 | 0.0370 |
| 272 | 11 | rs7112925 | 66,582,736 | RHOD | C | T | 0.0238 | 0.0030 |  | 0.0046 | 0.1037 |  | -0.0055 | 0.0180 |  | -0.0575 | 0.0188 |  | -0.0004 | 0.0178 |
| 273 | 11 | rs2510396 | 68,174,228 | GAL | C | G | 0.0289 | 0.0040 |  | -0.0557 | 0.0744 |  | 0.0091 | 0.0249 |  | -0.0341 | 0.0289 |  | -0.0148 | 0.0254 |
| 274 | 11 | rs606452 | 74,953,826 | SERPINH1 | A | C | 0.0429 | 0.0040 |  | 0.0489 | 0.0732 |  | 0.0243 | 0.0261 |  | -0.0326 | 0.0277 |  | -0.0108 | 0.0265 |
| 275 | 11 | rs632124 | 118,118,445 | DDX6 | A | T | 0.0226 | 0.0030 |  | -0.0022 | 0.0516 |  | -0.0027 | 0.0175 |  | -0.0061 | 0.0200 |  | 0.0335 | 0.0267 |
| 276 | 11 | rs10790381 | 119,762,705 | ARHGEF12 | A | G | 0.0270 | 0.0040 |  | 0.0780 | 0.0680 |  | 0.0137 | 0.0232 |  | -0.0055 | 0.0263 |  | 0.0046 | 0.0224 |
| 277 | 11 | rs1461503 | 122,350,285 | BSX | C | A | 0.0183 | 0.0030 |  | . | . |  | -0.0198 | 0.0174 |  | -0.0283 | 0.0194 |  | 0.0033 | 0.0171 |
| 278 | 11 | rs11221442 | 128,082,834 | FLI1 | G | C | 0.0267 | 0.0040 |  | -0.0355 | 0.0577 |  | -0.0143 | 0.0218 |  | 0.0105 | 0.0229 |  | -0.0246 | 0.0218 |
| 279 | 12 | rs11612228 | 447,245 | B4GALNT3 | T | C | 0.0200 | 0.0030 |  | 0.1321 | 0.0552 |  | -0.0118 | 0.0186 |  | -0.0097 | 0.0214 |  | -0.0042 | 0.0202 |
| 280 | 12 | rs2856321 | 11,747,040 | ETV6 | G | A | 0.0306 | 0.0030 |  | 0.0554 | 0.0545 |  | 0.0083 | 0.0183 |  | 0.0489 | 0.0188 |  | -0.0127 | 0.0178 |
| 281 | 12 | rs1420023 | 12,767,378 | CDKN1B | C | G | 0.0282 | 0.0050 |  | 0.2102 | 0.0792 |  | 0.0236 | 0.0296 |  | 0.0148 | 0.0313 |  | -0.0126 | 0.0299 |
| 282 | 12 | rs12228415 | 14,411,968 | ATF7IP | G | A | 0.0152 | 0.0030 |  | 0.0243 | 0.0510 |  | 0.0025 | 0.0176 |  | -0.0040 | 0.0201 |  | -0.0156 | 0.0171 |
| 283 | 12 | rs10770705 | 20,748,734 | SLCO1C1 | A | C | 0.0299 | 0.0030 |  | 0.0596 | 0.0559 |  | -0.0285 | 0.0188 |  | 0.0287 | 0.0215 |  | 0.0233 | 0.0186 |
| 284 | 12 | rs11047239 | 24,099,047 | SOX5 | G | C | 0.0233 | 0.0030 |  | 0.0080 | 0.0570 |  | 0.0016 | 0.0190 |  | 0.0139 | 0.0201 |  | 0.0179 | 0.0188 |
| 285 | 12 | rs11049611 | 28,491,511 | CCDC91 | C | T | 0.0381 | 0.0030 |  | 0.0040 | 0.0566 |  | 0.0822 | 0.0190 |  | -0.0144 | 0.0216 |  | 0.0161 | 0.0186 |
| 286 | 12 | rs10880969 | 45,113,290 | SLC38A2 | C | T | 0.0238 | 0.0030 |  | 0.0039 | 0.0568 |  | 0.0187 | 0.0199 |  | 0.0035 | 0.0210 |  | -0.0140 | 0.0186 |
| 287 | 12 | rs2306694 | 54,966,903 | CS | G | A | 0.0458 | 0.0060 |  | 0.0538 | 0.1154 |  | -0.0548 | 0.0362 |  | -0.0112 | 0.0400 |  | 0.0431 | 0.0336 |
| 288 | 12 | rs10877030 | 56,542,981 | CTDSP2 | T | G | 0.0226 | 0.0030 |  | 0.0032 | 0.0561 |  | 0.0190 | 0.0187 |  | 0.0336 | 0.0214 |  | -0.0255 | 0.0183 |
| 289 | 12 | rs17122659 | 58,243,190 | SLC16A7 | G | A | 0.0307 | 0.0050 |  | . | . |  | 0.0124 | 0.0294 |  | 0.0315 | 0.0333 |  | -0.0041 | 0.0288 |
| 290 | 12 | rs8756 | 64,646,019 | HMGA2 | C | A | 0.0585 | 0.0030 |  | -0.0016 | 0.0515 |  | -0.0151 | 0.0173 |  | -0.0164 | 0.0214 |  | 0.0093 | 0.0171 |
| 291 | 12 | rs10748128 | 68,113,925 | FRS2 | T | G | 0.0377 | 0.0030 |  | -0.0173 | 0.0534 |  | 0.0062 | 0.0197 |  | 0.0245 | 0.0228 |  | 0.0086 | 0.0182 |
| 292 | 12 | rs17783015 | 88,755,517 | ATP2B1 | C | T | 0.0229 | 0.0040 |  | -0.0221 | 0.0707 |  | 0.0054 | 0.0242 |  | 0.0438 | 0.0265 |  | -0.0423 | 0.0235 |
| 293 | 12 | rs3825199 | 92,501,085 | SOCS2 | G | A | 0.0512 | 0.0040 |  | 0.0894 | 0.0638 |  | 0.0042 | 0.0207 |  | -0.0412 | 0.0258 |  | -0.0126 | 0.0203 |
| 294 | 12 | rs7971536 | 100,897,919 | CCDC53 | T | A | 0.0285 | 0.0030 |  | 0.0905 | 0.0507 |  | -0.0063 | 0.0183 |  | -0.0054 | 0.0184 |  | 0.0276 | 0.0279 |
| 295 | 12 | rs2164747 | 102,868,966 | HSP90B1 | G | A | 0.0287 | 0.0050 |  | . | . |  | 0.0059 | 0.0282 |  | 0.0786 | 0.0300 |  | 0.1167 | 0.0302 |
| 296 | 12 | rs2888893 | 105,862,761 | C12orf23 | C | T | 0.0173 | 0.0030 |  | 0.0600 | 0.0511 |  | 0.0037 | 0.0176 |  | -0.0267 | 0.0180 |  | 0.0165 | 0.0188 |
| 297 | 12 | rs11616067 | 114,877,557 | MED13L | A | G | 0.0205 | 0.0040 |  | 0.0365 | 0.0611 |  | 0.0310 | 0.0211 |  | -0.0124 | 0.0239 |  | 0.0055 | 0.0204 |
| 298 | 12 | rs497273 | 119,689,065 | SPPL3 | C | G | 0.0176 | 0.0030 |  | -0.0285 | 0.0546 |  | 0.0117 | 0.0180 |  | -0.0146 | 0.0221 |  | -0.0103 | 0.0198 |
| 299 | 12 | rs11835818 | 120,979,192 | BCL7A | C | T | 0.0216 | 0.0030 |  | -0.0335 | 0.0514 |  | -0.0173 | 0.0174 |  | 0.0204 | 0.0247 |  | 0.0024 | 0.0170 |
| 300 | 12 | rs7980687 | 122,388,664 | SBNO1 | A | G | 0.0394 | 0.0040 |  | 0.0850 | 0.0633 |  | -0.0335 | 0.0224 |  | 0.0106 | 0.0354 |  | -0.0086 | 0.0214 |
| 301 | 13 | rs1199734 | 20,468,246 | LATS2 | G | T | 0.0218 | 0.0040 |  | -0.0822 | 0.0687 |  | 0.0234 | 0.0229 |  | -0.0113 | 0.0274 |  | 0.0099 | 0.0229 |
| 302 | 13 | rs11618507 | 29,070,751 | SLC7A1 | T | G | 0.0228 | 0.0040 |  | . | . |  | 0.0326 | 0.0225 |  | 0.0334 | 0.0240 |  | -0.0142 | 0.0232 |
| 303 | 13 | rs12323101 | 32,041,406 | PDS5B | A | G | 0.0205 | 0.0030 |  | 0.0209 | 0.0536 |  | 0.0092 | 0.0178 |  | 0.0326 | 0.0220 |  | -0.0064 | 0.0178 |
| 304 | 13 | rs6561319 | 46,010,121 | LRCH1 | A | C | 0.0209 | 0.0030 |  | 0.0775 | 0.0530 |  | 0.0010 | 0.0182 |  | 0.0411 | 0.0191 |  | -0.0408 | 0.0181 |
| 305 | 13 | rs12871822 | 48,099,041 | CYSLTR2 | G | T | 0.0169 | 0.0030 |  | 0.0383 | 0.0549 |  | -0.0100 | 0.0184 |  | 0.0284 | 0.0205 |  | 0.0050 | 0.0185 |
| 306 | 13 | rs3118905 | 50,003,335 | DLEU7 | G | A | 0.0578 | 0.0030 |  | -0.0337 | 0.0573 |  | 0.0073 | 0.0190 |  | -0.0059 | 0.0199 |  | -0.0123 | 0.0191 |
| 307 | 13 | rs4883972 | 73,956,482 | KLF12 | C | G | 0.0186 | 0.0030 |  | 0.0583 | 0.0513 |  | -0.0034 | 0.0176 |  | 0.0144 | 0.0215 |  | -0.0050 | 0.0279 |
| 308 | 13 | rs3818416 | 77,372,469 | EDNRB | C | A | 0.0211 | 0.0040 |  | 0.0010 | 0.0579 |  | 0.0277 | 0.0203 |  | -0.0343 | 0.0238 |  | 0.0289 | 0.0206 |
| 309 | 13 | rs11616380 | 79,603,316 | SPRY2 | T | G | 0.0188 | 0.0030 |  | -0.0216 | 0.0579 |  | -0.0058 | 0.0193 |  | -0.0146 | 0.0221 |  | -0.0545 | 0.0214 |
| 310 | 13 | rs7319045 | 90,822,575 | GPC5 | A | G | 0.0236 | 0.0030 |  | 0.0275 | 0.0522 |  | -0.0151 | 0.0180 |  | 0.0197 | 0.0187 |  | -0.0039 | 0.0180 |
| 311 | 13 | rs7985356 | 114,045,564 | CDC16 | T | A | 0.0227 | 0.0030 |  | 0.0664 | 0.0600 |  | -0.0170 | 0.0204 |  | -0.0015 | 0.0230 |  | -0.0108 | 0.0206 |
| 312 | 14 | rs17792664 | 20,960,523 | CHD8 | G | C | 0.0324 | 0.0040 |  | 0.2151 | 0.0793 |  | -0.0015 | 0.0253 |  | -0.0261 | 0.0304 |  | -0.0241 | 0.0252 |
| 313 | 14 | rs8017130 | 22,828,996 | HOMEZ | G | A | 0.0233 | 0.0030 |  | -0.0047 | 0.0553 |  | 0.0350 | 0.0198 |  | 0.0101 | 0.0238 |  | 0.0060 | 0.0184 |
| 314 | 14 | rs1950500 | 23,900,690 | NFATC4 | T | C | 0.0310 | 0.0030 |  | 0.0512 | 0.0576 |  | 0.0134 | 0.0191 |  | -0.0132 | 0.0219 |  | 0.0271 | 0.0187 |
| 315 | 14 | rs12435366 | 34,908,140 | NFKBIA | C | T | 0.0233 | 0.0040 |  | . | . |  | 0.0203 | 0.0206 |  | -0.0394 | 0.0213 |  | 0.0087 | 0.0201 |
| 316 | 14 | rs10131337 | 36,214,267 | PAX9 | T | C | 0.0269 | 0.0040 |  | . | . |  | 0.0059 | 0.0221 |  | 0.0099 | 0.0252 |  | 0.0232 | 0.0221 |
| 317 | 14 | rs8006657 | 54,314,899 | SAMD4A | G | A | 0.0219 | 0.0030 |  | 0.1192 | 0.1072 |  | 0.0190 | 0.0181 |  | -0.0258 | 0.0224 |  | 0.0535 | 0.0177 |
| 318 | 14 | rs11624136 | 58,758,573 | DAAM1 | A | G | 0.0176 | 0.0030 |  | -0.0449 | 0.0509 |  | 0.0089 | 0.0173 |  | -0.0215 | 0.0182 |  | -0.0011 | 0.0171 |
| 319 | 14 | rs2093210 | 60,027,032 | C14orf39 | C | T | 0.0389 | 0.0030 |  | -0.0012 | 0.0517 |  | 0.0201 | 0.0185 |  | -0.0087 | 0.0193 |  | -0.0021 | 0.0182 |
| 320 | 14 | rs2781373 | 64,637,968 | MAX | G | A | 0.0208 | 0.0030 |  | 0.0563 | 0.0533 |  | 0.0168 | 0.0178 |  | 0.0391 | 0.0187 |  | 0.0154 | 0.0176 |
| 321 | 14 | rs1980850 | 67,716,941 | RAD51L1 | G | A | 0.0301 | 0.0040 |  | -0.0009 | 0.0646 |  | -0.0402 | 0.0240 |  | -0.0030 | 0.0268 |  | -0.0237 | 0.0229 |
| 322 | 14 | rs2058092 | 73,002,719 | NUMB | T | C | 0.0172 | 0.0030 |  | -0.0315 | 0.0518 |  | 0.0052 | 0.0178 |  | 0.0126 | 0.0198 |  | -0.0236 | 0.0174 |
| 323 | 14 | rs862034 | 74,060,499 | LTBP2 | G | A | 0.0279 | 0.0030 |  | -0.1663 | 0.0539 |  | 0.0297 | 0.0181 |  | -0.0132 | 0.0206 |  | -0.0104 | 0.0176 |
| 324 | 14 | rs7154721 | 91,497,101 | TRIP11 | T | C | 0.0270 | 0.0030 |  | 0.0049 | 0.0515 |  | -0.0048 | 0.0175 |  | -0.0287 | 0.0181 |  | 0.0064 | 0.0172 |
| 325 | 14 | rs12882130 | 102,948,527 | MARK3 | C | G | 0.0251 | 0.0030 |  | 0.0620 | 0.0559 |  | 0.0058 | 0.0190 |  | -0.0504 | 0.0200 |  | -0.0255 | 0.0186 |
| 326 | 15 | rs10152739 | 36,271,158 | SPRED1 | T | A | 0.0219 | 0.0030 |  | -0.1494 | 0.0576 |  | -0.0027 | 0.0200 |  | -0.0450 | 0.0234 |  | -0.0157 | 0.0201 |
| 327 | 15 | rs316618 | 39,583,790 | LTK | T | A | 0.0259 | 0.0040 |  | -0.0778 | 0.0623 |  | 0.0105 | 0.0221 |  | 0.0526 | 0.0244 |  | -0.0020 | 0.0209 |
| 328 | 15 | rs1036477 | 46,702,218 | FBN1 | A | G | 0.0319 | 0.0050 |  | 0.0043 | 0.0799 |  | -0.0394 | 0.0279 |  | -0.0351 | 0.0352 |  | -0.0089 | 0.0276 |
| 329 | 15 | rs16964211 | 49,317,787 | CYP19A1 | G | A | 0.0570 | 0.0070 |  | -0.1194 | 0.1147 |  | -0.0398 | 0.0409 |  | -0.0103 | 0.0483 |  | 0.0087 | 0.0386 |
| 330 | 15 | rs7177711 | 60,167,263 | FAM148A | A | G | 0.0213 | 0.0030 |  | 0.1049 | 0.0513 |  | -0.0169 | 0.0174 |  | -0.0171 | 0.0180 |  | -0.0123 | 0.0173 |
| 331 | 15 | rs7162825 | 61,226,239 | LACTB | T | C | 0.0162 | 0.0030 |  | -0.1157 | 0.0524 |  | -0.0088 | 0.0175 |  | 0.0148 | 0.0180 |  | 0.0050 | 0.0171 |
| 332 | 15 | rs17264185 | 64,784,141 | SMAD6 | G | A | 0.0203 | 0.0030 |  | -0.0382 | 0.0584 |  | -0.0116 | 0.0201 |  | 0.0327 | 0.0243 |  | 0.0183 | 0.0196 |
| 333 | 15 | rs975210 | 68,151,406 | TLE3 | A | G | 0.0348 | 0.0040 |  | . | . |  | -0.0077 | 0.0247 |  | -0.0227 | 0.0247 |  | 0.0044 | 0.0251 |
| 334 | 15 | rs12904334 | 70,629,759 | ARIH1 | A | G | 0.0843 | 0.0130 |  | -0.0998 | 0.2278 |  | 0.1337 | 0.0852 |  | 0.0916 | 0.0959 |  | -0.0651 | 0.0755 |
| 335 | 15 | rs5742915 | 72,123,686 | PML | C | T | 0.0350 | 0.0030 |  | . | . |  | -0.0390 | 0.0181 |  | 0.0144 | 0.0201 |  | 0.0155 | 0.0171 |
| 336 | 15 | rs16968242 | 74,527,274 | SCAPER | G | C | 0.0350 | 0.0060 |  | 0.1290 | 0.0937 |  | -0.0902 | 0.0336 |  | 0.0281 | 0.0409 |  | 0.0391 | 0.0334 |
| 337 | 15 | rs17349981 | 80,018,975 | MEX3B | A | T | 0.0249 | 0.0040 |  | -0.0051 | 0.0791 |  | -0.0235 | 0.0248 |  | 0.0123 | 0.0294 |  | 0.0086 | 0.0252 |
| 338 | 15 | rs2257011 | 82,057,149 | SH3GL3 | T | G | 0.0435 | 0.0030 |  | . | . |  | 0.0092 | 0.0184 |  | 0.0373 | 0.0199 |  | 0.0157 | 0.0174 |
| 339 | 15 | rs11855014 | 83,529,838 | PDE8A | G | A | 0.0217 | 0.0030 |  | 0.0897 | 0.0546 |  | 0.0166 | 0.0193 |  | 0.0324 | 0.0210 |  | 0.0181 | 0.0210 |
| 340 | 15 | rs2280470 | 87,196,630 | ACAN | A | G | 0.0436 | 0.0030 |  | 0.0341 | 0.0554 |  | -0.0175 | 0.0187 |  | -0.0081 | 0.0228 |  | 0.0089 | 0.0180 |
| 341 | 15 | rs7181724 | 92,352,611 | MCTP2 | G | A | 0.0204 | 0.0030 |  | 0.0458 | 0.0522 |  | 0.0204 | 0.0183 |  | -0.0213 | 0.0219 |  | -0.0191 | 0.0186 |
| 342 | 15 | rs2871865 | 97,012,419 | IGF1R | C | G | 0.0616 | 0.0050 |  | 0.1116 | 0.0775 |  | -0.0337 | 0.0281 |  | 0.0009 | 0.0358 |  | -0.0164 | 0.0304 |
| 343 | 15 | rs4548838 | 98,578,713 | ADAMTS17 | T | C | 0.0330 | 0.0030 |  | . | . |  | -0.0261 | 0.0177 |  | -0.0035 | 0.0192 |  | 0.0106 | 0.0189 |
| 344 | 16 | rs11648796 | 732,191 | NARFL | G | A | 0.0332 | 0.0040 |  | 0.1177 | 0.0671 |  | 0.0689 | 0.0230 |  | -0.0231 | 0.0275 |  | -0.0133 | 0.0248 |
| 345 | 16 | rs26868 | 2,189,377 | CASKIN1 | A | T | 0.0290 | 0.0030 |  | -0.0285 | 0.0519 |  | -0.0069 | 0.0187 |  | -0.0288 | 0.0191 |  | 0.0155 | 0.0281 |
| 346 | 16 | rs1659127 | 14,295,806 | MKL2 | A | G | 0.0297 | 0.0030 |  | 0.0306 | 0.0553 |  | 0.0199 | 0.0200 |  | 0.0128 | 0.0234 |  | -0.0003 | 0.0186 |
| 347 | 16 | rs2023693 | 20,787,541 | DCUN1D3 | G | A | 0.0165 | 0.0030 |  | -0.0116 | 0.0512 |  | -0.0337 | 0.0175 |  | -0.0079 | 0.0216 |  | 0.0058 | 0.0172 |
| 348 | 16 | rs11642612 | 29,937,696 | FLJ25404 | C | A | 0.0164 | 0.0030 |  | 0.0887 | 0.0519 |  | 0.0466 | 0.0177 |  | -0.0099 | 0.0219 |  | 0.0138 | 0.0174 |
| 349 | 16 | rs4785393 | 48,816,984 | PAPD5 | G | A | 0.0228 | 0.0040 |  | 0.0666 | 0.0629 |  | 0.0051 | 0.0232 |  | 0.0027 | 0.0284 |  | -0.0202 | 0.0229 |
| 350 | 16 | rs8058684 | 52,072,619 | RBL2 | A | G | 0.0208 | 0.0030 |  | 0.0608 | 0.0548 |  | -0.0047 | 0.0193 |  | 0.0560 | 0.0232 |  | 0.0115 | 0.0188 |
| 351 | 16 | rs1966913 | 65,941,727 | LRRC36 | A | T | 0.0440 | 0.0070 |  | 0.0491 | 0.1216 |  | 0.0382 | 0.0436 |  | 0.0816 | 0.0489 |  | -0.0469 | 0.0435 |
| 352 | 16 | rs3790086 | 68,445,208 | WWP2 | C | G | 0.0231 | 0.0030 |  | 0.0251 | 0.0518 |  | -0.0167 | 0.0172 |  | -0.0125 | 0.0214 |  | -0.0415 | 0.0268 |
| 353 | 16 | rs217181 | 70,671,503 | HPR | T | C | 0.0238 | 0.0040 |  | -0.0734 | 0.0651 |  | -0.0202 | 0.0225 |  | -0.0741 | 0.0253 |  | 0.0060 | 0.0220 |
| 354 | 16 | rs11640018 | 73,885,809 | CFDP1 | C | T | 0.0188 | 0.0030 |  | 0.0301 | 0.0530 |  | -0.0349 | 0.0193 |  | 0.0172 | 0.0224 |  | -0.0340 | 0.0178 |
| 355 | 16 | rs6420435 | 80,741,702 | MPHOSPH6 | A | C | 0.0233 | 0.0040 |  | -0.0152 | 0.0614 |  | -0.0279 | 0.0215 |  | 0.0523 | 0.0216 |  | 0.0396 | 0.0208 |
| 356 | 16 | rs2326458 | 83,545,180 | ZDHHC7 | C | A | 0.0219 | 0.0040 |  | 0.0794 | 0.0581 |  | -0.0084 | 0.0206 |  | -0.0045 | 0.0242 |  | 0.0357 | 0.0192 |
| 357 | 16 | rs4843367 | 84,975,391 | FOXF1 | C | T | 0.0191 | 0.0030 |  | . | . |  | 0.0231 | 0.0184 |  | 0.0038 | 0.0210 |  | 0.0020 | 0.0180 |
| 358 | 16 | rs8052560 | 87,304,743 | C16orf84 | A | C | 0.0360 | 0.0040 |  | -0.0334 | 0.0690 |  | 0.0144 | 0.0270 |  | -0.0015 | 0.0260 |  | 0.0197 | 0.0266 |
| 359 | 17 | rs870183 | 546,561 | VPS53 | G | A | 0.0170 | 0.0030 |  | . | . |  | 0.0227 | 0.0172 |  | -0.0613 | 0.0180 |  | -0.0083 | 0.0173 |
| 360 | 17 | rs9217 | 7,303,812 | ZBTB4 | C | T | 0.0279 | 0.0030 |  | 0.0383 | 0.0537 |  | 0.0029 | 0.0181 |  | 0.0045 | 0.0210 |  | -0.0163 | 0.0179 |
| 361 | 17 | rs8069300 | 11,924,957 | MAP2K4 | G | C | 0.0158 | 0.0030 |  | 0.0662 | 0.0505 |  | 0.0622 | 0.0173 |  | 0.0014 | 0.0212 |  | -0.0025 | 0.0267 |
| 362 | 17 | rs4640244 | 21,224,816 | KCNJ12 | A | G | 0.0254 | 0.0030 |  | -0.0326 | 0.1059 |  | -0.0192 | 0.0191 |  | 0.0140 | 0.0227 |  | -0.0153 | 0.0193 |
| 363 | 17 | rs3809790 | 24,979,666 | SSH2 | C | T | 0.0160 | 0.0030 |  | 0.0191 | 0.0511 |  | -0.0081 | 0.0174 |  | -0.0211 | 0.0213 |  | 0.0407 | 0.0174 |
| 364 | 17 | rs3760318 | 26,271,841 | CENTA2 | G | A | 0.0407 | 0.0030 |  | 0.0688 | 0.0527 |  | 0.0437 | 0.0179 |  | 0.0138 | 0.0206 |  | -0.0183 | 0.0174 |
| 365 | 17 | rs2338115 | 34,183,104 | PIP4K2B | T | C | 0.0227 | 0.0030 |  | -0.0688 | 0.0521 |  | -0.0204 | 0.0173 |  | 0.0242 | 0.0195 |  | 0.0055 | 0.0170 |
| 366 | 17 | rs584828 | 35,852,756 | IGFBP4 | C | T | 0.0254 | 0.0030 |  | 0.0187 | 0.0525 |  | -0.0061 | 0.0182 |  | -0.0081 | 0.0204 |  | 0.0009 | 0.0176 |
| 367 | 17 | rs9766 | 38,106,367 | EZH1 | A | G | 0.0209 | 0.0030 |  | -0.0057 | 0.0513 |  | -0.0232 | 0.0173 |  | 0.0392 | 0.0181 |  | 0.0090 | 0.0172 |
| 368 | 17 | rs4986172 | 40,571,807 | ACBD4 | C | T | 0.0344 | 0.0030 |  | 0.0268 | 0.0533 |  | -0.0040 | 0.0184 |  | 0.0185 | 0.0199 |  | -0.0377 | 0.0179 |
| 369 | 17 | rs199515 | 42,211,804 | WNT3 | C | G | 0.0161 | 0.0040 |  | 0.0471 | 0.0615 |  | 0.0584 | 0.0222 |  | 0.0169 | 0.0245 |  | 0.0035 | 0.0224 |
| 370 | 17 | rs318095 | 44,329,733 | ATP5G1 | T | C | 0.0240 | 0.0030 |  | 0.0096 | 0.0506 |  | -0.0194 | 0.0172 |  | 0.0080 | 0.0189 |  | -0.0007 | 0.0169 |
| 371 | 17 | rs4605213 | 46,599,746 | NME1-NME2/NME2 | C | G | 0.0184 | 0.0030 |  | 0.0153 | 0.0543 |  | 0.0418 | 0.0188 |  | -0.0222 | 0.0198 |  | -0.0052 | 0.0186 |
| 372 | 17 | rs1401795 | 52,194,651 | C17orf67 | A | G | 0.0295 | 0.0030 |  | 0.0349 | 0.0510 |  | -0.0266 | 0.0188 |  | 0.0461 | 0.0215 |  | -0.0282 | 0.0180 |
| 373 | 17 | rs2079795 | 56,851,431 | C17orf82 | T | C | 0.0445 | 0.0030 |  | -0.0179 | 0.0543 |  | 0.0416 | 0.0186 |  | -0.0224 | 0.0228 |  | 0.0102 | 0.0183 |
| 374 | 17 | rs2854207 | 59,300,839 | CSH2 | G | C | 0.0455 | 0.0030 |  | -0.0475 | 0.0578 |  | -0.0012 | 0.0197 |  | -0.0023 | 0.0212 |  | 0.0132 | 0.0210 |
| 375 | 17 | rs3923086 | 60,979,950 | AXIN2 | C | A | 0.0235 | 0.0030 |  | . | . |  | -0.0149 | 0.0185 |  | -0.0008 | 0.0222 |  | 0.0010 | 0.0172 |
| 376 | 17 | rs2072268 | 63,814,947 | ARSG | G | A | 0.0203 | 0.0030 |  | . | . |  | 0.0197 | 0.0181 |  | -0.0056 | 0.0198 |  | 0.0077 | 0.0170 |
| 377 | 17 | rs11867479 | 65,601,802 | KCNJ16 | T | C | 0.0261 | 0.0030 |  | -0.0062 | 0.0542 |  | 0.0357 | 0.0186 |  | -0.0128 | 0.0199 |  | -0.0349 | 0.0186 |
| 378 | 17 | rs10083886 | 67,434,950 | SOX9 | T | C | 0.0192 | 0.0030 |  | 0.0256 | 0.0562 |  | -0.0157 | 0.0195 |  | 0.0501 | 0.0239 |  | -0.0017 | 0.0193 |
| 379 | 17 | rs2117563 | 70,880,580 | GRB2 | G | A | 0.0240 | 0.0040 |  | 0.0958 | 0.0640 |  | 0.0219 | 0.0224 |  | 0.0228 | 0.0295 |  | 0.0185 | 0.0245 |
| 380 | 17 | rs1552173 | 74,230,437 | PSCD1 | C | T | 0.0181 | 0.0030 |  | -0.0264 | 0.0515 |  | 0.0116 | 0.0173 |  | -0.0098 | 0.0220 |  | -0.0088 | 0.0174 |
| 381 | 17 | rs4239020 | 77,769,930 | CCDC57 | C | T | 0.0214 | 0.0030 |  | -0.0859 | 0.0555 |  | -0.0052 | 0.0188 |  | 0.0032 | 0.0230 |  | -0.0117 | 0.0182 |
| 382 | 18 | rs888403 | 2,756,938 | SMCHD1 | G | A | 0.0189 | 0.0030 |  | . | . |  | 0.0276 | 0.0189 |  | 0.0051 | 0.0191 |  | -0.0370 | 0.0183 |
| 383 | 18 | rs692964 | 13,084,132 | CEP192 | G | A | 0.0190 | 0.0030 |  | -0.0715 | 0.0538 |  | -0.0045 | 0.0177 |  | 0.0416 | 0.0206 |  | -0.0106 | 0.0175 |
| 384 | 18 | rs14062 | 17,704,301 | MIB1 | G | A | 0.0179 | 0.0030 |  | -0.0448 | 0.0542 |  | 0.0064 | 0.0185 |  | 0.0028 | 0.0209 |  | 0.0051 | 0.0179 |
| 385 | 18 | rs4369779 | 18,989,406 | CABLES1 | C | T | 0.0561 | 0.0040 |  | -0.0269 | 0.0652 |  | -0.0181 | 0.0213 |  | 0.0406 | 0.0263 |  | 0.0149 | 0.0210 |
| 386 | 18 | rs9967417 | 45,213,498 | DYM | G | C | 0.0404 | 0.0030 |  | 0.0603 | 0.0521 |  | 0.0124 | 0.0176 |  | 0.0105 | 0.0199 |  | -0.0444 | 0.0229 |
| 387 | 18 | rs11152213 | 56,003,928 | MC4R | C | A | 0.0250 | 0.0040 |  | 0.0358 | 0.0602 |  | -0.0251 | 0.0208 |  | -0.0209 | 0.0249 |  | 0.0313 | 0.0203 |
| 388 | 18 | rs8097893 | 73,112,043 | GALR1 | A | G | 0.0424 | 0.0070 |  | -0.0873 | 0.1443 |  | 0.0192 | 0.0422 |  | -0.0309 | 0.0498 |  | -0.0263 | 0.0410 |
| 389 | 18 | rs11659752 | 75,323,850 | NFATC1 | T | G | 0.0244 | 0.0030 |  | -0.0918 | 0.0561 |  | 0.0024 | 0.0194 |  | -0.0026 | 0.0238 |  | 0.0220 | 0.0184 |
| 390 | 19 | rs11880992 | 2,127,403 | DOT1L | A | G | 0.0330 | 0.0030 |  | 0.0341 | 0.0522 |  | -0.0218 | 0.0178 |  | 0.0274 | 0.0217 |  | -0.0279 | 0.0173 |
| 391 | 19 | rs2074977 | 3,385,028 | NFIC | C | A | 0.0292 | 0.0030 |  | 0.0103 | 0.0538 |  | -0.0086 | 0.0191 |  | -0.0310 | 0.0224 |  | -0.0147 | 0.0180 |
| 392 | 19 | rs2123731 | 4,880,473 | UHRF1 | A | G | 0.0233 | 0.0040 |  | -0.0342 | 0.0566 |  | 0.0305 | 0.0199 |  | -0.0210 | 0.0226 |  | 0.0110 | 0.0198 |
| 393 | 19 | rs891088 | 7,135,762 | INSR | G | A | 0.0291 | 0.0030 |  | 0.1206 | 0.0583 |  | -0.0074 | 0.0199 |  | -0.0389 | 0.0243 |  | 0.0127 | 0.0193 |
| 394 | 19 | rs4072910 | 8,550,031 | ADAMTS10 | G | C | 0.0320 | 0.0040 |  | -0.0562 | 0.0514 |  | 0.0282 | 0.0234 |  | 0.0080 | 0.0229 |  | 0.0082 | 0.0329 |
| 395 | 19 | rs8102380 | 10,662,185 | ILF3 | G | A | 0.0204 | 0.0030 |  | -0.0158 | 0.0545 |  | -0.0138 | 0.0184 |  | -0.0106 | 0.0201 |  | -0.0296 | 0.0183 |
| 396 | 19 | rs7259684 | 12,047,611 | LOC729747 | G | A | 0.0354 | 0.0060 |  | -0.0092 | 0.0889 |  | -0.0244 | 0.0353 |  | -0.0125 | 0.0341 |  | 0.0278 | 0.0354 |
| 397 | 19 | rs8103068 | 17,383,869 | BST2 | T | C | 0.0311 | 0.0050 |  | -0.0208 | 0.0701 |  | -0.0352 | 0.0259 |  | -0.0328 | 0.0314 |  | 0.0360 | 0.0241 |
| 398 | 19 | rs8103992 | 19,526,643 | PBX4 | A | C | 0.0286 | 0.0040 |  | -0.0524 | 0.0612 |  | 0.0506 | 0.0215 |  | 0.0491 | 0.0271 |  | 0.0277 | 0.0218 |
| 399 | 19 | rs7253628 | 35,739,109 | ZNF536 | G | A | 0.0239 | 0.0040 |  | . | . |  | 0.0200 | 0.0233 |  | -0.0135 | 0.0265 |  | 0.0004 | 0.0235 |
| 400 | 19 | rs4802134 | 43,038,525 | SIPA1L3 | A | G | 0.0265 | 0.0040 |  | -0.0553 | 0.0599 |  | -0.0022 | 0.0235 |  | 0.0394 | 0.0227 |  | -0.0001 | 0.0206 |
| 401 | 19 | rs4803468 | 46,614,192 | BCKDHA | A | G | 0.0300 | 0.0030 |  | -0.0428 | 0.0518 |  | -0.0215 | 0.0184 |  | 0.0098 | 0.0219 |  | -0.0008 | 0.0193 |
| 402 | 19 | rs2682587 | 48,774,269 | XRCC1 | A | C | 0.0227 | 0.0040 |  | -0.0846 | 0.0665 |  | -0.0126 | 0.0223 |  | -0.0127 | 0.0271 |  | -0.0052 | 0.0218 |
| 403 | 19 | rs2059877 | 52,880,621 | GLTSCR1 | T | G | 0.0186 | 0.0030 |  | 0.0679 | 0.0576 |  | -0.0055 | 0.0199 |  | 0.0027 | 0.0219 |  | 0.0185 | 0.0193 |
| 404 | 20 | rs7273787 | 4,046,567 | SMOX | G | A | 0.0218 | 0.0030 |  | 0.0369 | 0.0533 |  | 0.0162 | 0.0184 |  | -0.0126 | 0.0208 |  | 0.0200 | 0.0184 |
| 405 | 20 | rs1884897 | 6,560,832 | BMP2 | A | G | 0.0443 | 0.0030 |  | 0.1484 | 0.0525 |  | 0.0166 | 0.0179 |  | -0.0422 | 0.0203 |  | 0.0069 | 0.0177 |
| 406 | 20 | rs6080830 | 17,719,113 | BANF2 | A | G | 0.0162 | 0.0030 |  | 0.0067 | 0.0517 |  | -0.0240 | 0.0174 |  | -0.0109 | 0.0203 |  | -0.0081 | 0.0171 |
| 407 | 20 | rs7261425 | 20,016,635 | C20orf26 | C | G | 0.0214 | 0.0030 |  | 0.0172 | 0.0569 |  | 0.0049 | 0.0198 |  | -0.0251 | 0.0203 |  | 0.0071 | 0.0196 |
| 408 | 20 | rs1074683 | 31,768,314 | PXMP4 | C | G | 0.0443 | 0.0030 |  | -0.0765 | 0.0591 |  | -0.0052 | 0.0202 |  | -0.0135 | 0.0233 |  | -0.0374 | 0.0199 |
| 409 | 20 | rs2425163 | 33,896,084 | PHF20 | G | A | 0.0579 | 0.0040 |  | -0.0013 | 0.0647 |  | 0.0422 | 0.0228 |  | -0.0798 | 0.0247 |  | 0.0094 | 0.0219 |
| 410 | 20 | rs4812586 | 34,978,087 | SAMHD1 | A | G | 0.0302 | 0.0040 |  | -0.0541 | 0.0733 |  | -0.0614 | 0.0242 |  | 0.0242 | 0.0258 |  | 0.0355 | 0.0236 |
| 411 | 20 | rs2224538 | 37,985,492 | MAFB | T | C | 0.0174 | 0.0030 |  | -0.1848 | 0.0534 |  | 0.0162 | 0.0182 |  | 0.0022 | 0.0208 |  | -0.0052 | 0.0179 |
| 412 | 20 | rs17450430 | 47,205,671 | STAU1 | T | A | 0.0352 | 0.0030 |  | -0.0454 | 0.0631 |  | -0.0197 | 0.0206 |  | 0.0190 | 0.0239 |  | -0.0226 | 0.0231 |
| 413 | 20 | rs1326023 | 54,275,785 | MC3R | A | G | 0.0238 | 0.0030 |  | -0.0979 | 0.0549 |  | -0.0245 | 0.0193 |  | 0.0189 | 0.0206 |  | 0.0104 | 0.0186 |
| 414 | 20 | rs2057291 | 56,905,438 | GNAS | A | G | 0.0202 | 0.0030 |  | 0.0343 | 0.0548 |  | 0.0200 | 0.0204 |  | 0.0202 | 0.0213 |  | 0.0218 | 0.0183 |
| 415 | 20 | rs6061231 | 60,390,312 | RPS21 | C | A | 0.0209 | 0.0030 |  | 0.1574 | 0.0578 |  | 0.0231 | 0.0192 |  | -0.0486 | 0.0208 |  | 0.0105 | 0.0190 |
| 416 | 21 | rs2829941 | 26,130,806 | APP | T | G | 0.0166 | 0.0030 |  | 0.0200 | 0.0520 |  | -0.0168 | 0.0177 |  | 0.0167 | 0.0186 |  | 0.0142 | 0.0174 |
| 417 | 21 | rs2834442 | 34,612,656 | KCNE2 | A | T | 0.0239 | 0.0030 |  | 0.0765 | 0.0528 |  | 0.0111 | 0.0180 |  | 0.0292 | 0.0193 |  | 0.0074 | 0.0181 |
| 418 | 21 | rs2211866 | 38,609,977 | KCNJ15 | A | G | 0.0216 | 0.0030 |  | -0.0233 | 0.0526 |  | -0.0237 | 0.0177 |  | -0.0215 | 0.0195 |  | -0.0282 | 0.0172 |
| 419 | 21 | rs9977276 | 46,260,755 | COL6A1 | G | T | 0.0223 | 0.0040 |  | 0.0091 | 0.0611 |  | -0.0081 | 0.0207 |  | 0.0491 | 0.0236 |  | 0.0346 | 0.0208 |
| 420 | 22 | rs2413143 | 31,386,859 | SYN3 | C | T | 0.0341 | 0.0040 |  | . | . |  | -0.0427 | 0.0243 |  | -0.0515 | 0.0479 |  | -0.0147 | 0.0361 |
| 421 | 22 | rs7284476 | 36,459,278 | TRIOBP | A | G | 0.0177 | 0.0030 |  | 0.0038 | 0.0512 |  | 0.0084 | 0.0178 |  | 0.0488 | 0.0216 |  | -0.0101 | 0.0171 |
| 422 | 22 | rs738288 | 38,237,607 | SMCR7L | G | A | 0.0195 | 0.0030 |  | 0.0072 | 0.0522 |  | 0.0341 | 0.0178 |  | -0.0143 | 0.0199 |  | -0.0111 | 0.0170 |
| 423 | 22 | rs11090631 | 44,225,035 | RIBC2 | T | C | 0.0205 | 0.0040 |  | -0.0112 | 0.0618 |  | 0.0066 | 0.0229 |  | -0.0026 | 0.0243 |  | 0.0077 | 0.0215 |

Note: All variants are independent at an r^2^ < 0.5.
