# Supplementary material for: Association between Adult Height and Risk of Colorectal, Lung, and Prostate Cancer: Results from Meta-analyses of Prospective Studies and Mendelian Randomization Analyses
Source: PLoS Med. 2016 Sep 6;13(9):e1002118. doi: 10.1371/journal.pmed.1002118 (PMC5012582; doi:10.1371/journal.pmed.1002118)
Supplement: S6 Table — (DOCX) [file pmed.1002118.s011.docx]

**S6 Table.**  Random-effects summary estimate from published prospective studies of populations of European descent for the association between height (standardized to 10-cm increase) and multiple cancers

| **Cancer site** |  | **Meta-analysis**  **random-effects** | |
| --- | --- | --- | --- |
|  |  | **RR** | **95% CI** |
|  |  |  |  |
| **Breast:** |  |  |  |
| Overall |  | 1.17^a^ | 1.14, 1.20 |
| **Colorectal:** |  |  |  |
| Overall |  | 1.13 | 1.10, 1.16 |
| **Prostate:** |  |  |  |
| Overall |  | 1.08 | 1.05, 1.10 |
| Aggressive |  | 1.06 | 0.96, 1.16 |
| **Lung:** |  |  |  |
| Overall |  | 1.03 | 0.99, 1.07 |
| Adenocarcinoma |  | na | na |
| Squamous |  | na | na |

Note:

^a^ Results (OR and 99% CI) previously reported from Million Women’s Study
