# Supplementary material for: Association between Adult Height and Risk of Colorectal, Lung, and Prostate Cancer: Results from Meta-analyses of Prospective Studies and Mendelian Randomization Analyses
Source: PLoS Med. 2016 Sep 6;13(9):e1002118. doi: 10.1371/journal.pmed.1002118 (PMC5012582; doi:10.1371/journal.pmed.1002118)
Supplement: S7 Table — (DOCX) [file pmed.1002118.s012.docx]

**S7 Table.** Inverse-variance-weighted odds ratios and 95% CIs estimated using a fixed-effects meta-analysis model for the association between adult height and multiple cancers using Mendelian randomization stratified by sex for colorectal and lung cancers

| **Cancer site** |  | **Consortium**  **sample size^a^** | |  | **IV^b^** |  | **Males** | | |  | **Females** | | |
| --- | --- | --- | --- | --- | --- | --- | --- | --- | --- | --- | --- | --- | --- |
|  |  | **Cases** | **Controls** |  |  |  | **OR^c^** | **95% CI** | **p** |  | **OR^c^** | **95% CI** | **p** |
| **Colorectal:** |  |  |  |  |  |  |  |  |  |  |  |  |  |
| Overall |  | 5,100 | 4,831 |  | 153 |  | 1.17 | 0.79, 1.74 | 0.436 |  | 1.24 | 0.81, 1.90 | 0.327 |
| **Lung:** |  |  |  |  |  |  |  |  |  |  |  |  |  |
| Overall |  | 12,537 | 17,285 |  | 168 |  | 1.16 | 1.02, 1.33 | 0.024 |  | 1.15 | 1.00, 1.33 | 0.049 |
| Adenocarcinoma |  | 3,804 | 16,289 |  | 168 |  | 1.13 | 0.93, 1.38 | 0.216 |  | 1.13 | 0.92, 1.40 | 0.254 |
| Squamous |  | 3,546 | 16,434 |  | 168 |  | 1.09 | 0.88, 1.34 | 0.443 |  | 1.07 | 0.86, 1.34 | 0.539 |

Note:

^a^Summary sample sizes of studies included in the Genetic Associations and Mechanisms in Oncology (GAME-ON) consortium.

^b^The total number of single nucleotide polymorphisms (SNPs) used as the instrumental variable (IV).

^c^Fixed-effects OR represent 10 cm (≈3.94 in) increase in adult height per standard deviation derived from GIANT consortium [Lango Allen, 2010]
