# Supplementary material for: Association between Adult Height and Risk of Colorectal, Lung, and Prostate Cancer: Results from Meta-analyses of Prospective Studies and Mendelian Randomization Analyses
Source: PLoS Med. 2016 Sep 6;13(9):e1002118. doi: 10.1371/journal.pmed.1002118 (PMC5012582; doi:10.1371/journal.pmed.1002118)
Supplement: S8 Table — (DOCX) [file pmed.1002118.s013.docx]

**S8 Table.** Inverse-variance-weighted odds ratios and 95% CIs estimated using a fixed-effects meta-analysis model for the association between adult height and multiple cancers using Mendelian randomization analysis with updated GWAS data excluding SNPs with demonstrated pleiotropic effects

| **Cancer site** |  | **Consortium**  **sample size^a^** | |  | **Mendelian**  **randomization** | | | |
| --- | --- | --- | --- | --- | --- | --- | --- | --- |
|  |  | **Cases** | **Controls** |  | **IV^b^** | **OR^c^** | **95% CI** | **p** |
| **Breast:** |  |  |  |  |  |  |  |  |
| Overall |  | 16,003 | 46,525 |  | 392 | 1.19 | 1.06, 1.34 | 0.003 |
| **Colorectal:** |  |  |  |  |  |  |  |  |
| Overall |  | 5,100 | 4,831 |  | 319 | 1.65 | 1.17, 2.34 | 0.005 |
| **Prostate:** |  |  |  |  |  |  |  |  |
| Overall |  | 14,160 | 12,712 |  | 389 | 0.98 | 0.87, 1.11 | 0.790 |
| Aggressive |  | 4,446 | 12,724 |  | 389 | 0.95 | 0.80, 1.13 | 0.565 |
| **Lung:** |  |  |  |  |  |  |  |  |
| Overall |  | 12,537 | 17,285 |  | 392 | 1.10 | 0.98, 1.22 | 0.096 |
| Adenocarcinoma |  | 3,804 | 16,289 |  | 392 | 1.16 | 0.98, 1.36 | 0.081 |
| Squamous |  | 3,546 | 16,434 |  | 392 | 1.06 | 0.89, 1.26 | 0.506 |

Note:

^a^Summary sample sizes of studies included in the Genetic Associations and Mechanisms in Oncology (GAME-ON) consortium.

^b^The total number of single nucleotide polymorphisms (SNPs) used as the instrumental variable (IV). A total of 36 SNPs had known pleiotropic effects. However not all SNPs were available in each dataset, thus resulting in different numbers of exclusions for each cancer site.

^c^Fixed-effects OR represent 10 cm (≈3.94 in) increase in adult height per standard deviation derived from GIANT consortium [Wood, 2014]
