# Supplementary material for: Association between Adult Height and Risk of Colorectal, Lung, and Prostate Cancer: Results from Meta-analyses of Prospective Studies and Mendelian Randomization Analyses
Source: PLoS Med. 2016 Sep 6;13(9):e1002118. doi: 10.1371/journal.pmed.1002118 (PMC5012582; doi:10.1371/journal.pmed.1002118)
Supplement: S9 Table — (DOCX) [file pmed.1002118.s014.docx]

| **Cancer site** |  | **Consortium**  **sample size** | |  | **IV^b^** |  | **Egger**  **estimate^a^** | | |  | **Intercept from**  **Egger regression^b^** | |
| --- | --- | --- | --- | --- | --- | --- | --- | --- | --- | --- | --- | --- |
|  |  | **Cases** | **Controls** |  |  |  | **OR** | **95% CI** | **p** |  | **β_0_** | **p** |
| **Breast:** |  |  |  |  |  |  |  |  |  |  |  |  |
| Overall |  | 16,003 | 46,525 |  | 423 |  | 1.25 | 0.88, 1.78 | 0.21 |  | -0.0009 | 0.78 |
| **Colorectal:** |  |  |  |  |  |  |  |  |  |  |  |  |
| Overall |  | 5,100 | 4,831 |  | 346 |  | 0.92 | 0.37, 2.32 | 0.87 |  | 0.0108 | 0.22 |
| **Prostate:** |  |  |  |  |  |  |  |  |  |  |  |  |
| Overall |  | 14,160 | 12,712 |  | 419 |  | 1.27 | 0.88, 1.86 | 0.20 |  | -0.0045 | 0.22 |
| Aggressive |  | 4,446 | 12,724 |  | 419 |  | 0.91 | 0.56, 1.49 | 0.71 |  | 0.0016 | 0.75 |
| **Lung:** |  |  |  |  |  |  |  |  |  |  |  |  |
| Overall |  | 12,537 | 17,285 |  | 423 |  | 1.04 | 0.77, 1.39 | 0.82 |  | 0.0013 | 0.64 |
| Adenocarcinoma |  | 3,804 | 16,289 |  | 423 |  | 0.97 | 0.63, 1.49 | 0.88 |  | 0.0037 | 0.38 |
| Squamous |  | 3,546 | 16,434 |  | 423 |  | 1.03 | 0.65, 1.62 | 0.92 |  | 0.0007 | 0.87 |

^a^ Bias-reduced estimate for the potential causal effect.

^b^ Estimate of the average pleiotropic effect across the genetic variants.
